# Symmetry-Driven Phonon Confinement in 2D Halide Perovskites

**DOI:** 10.1021/acs.jpclett.6c01515

**Published:** 2026-06-14

**Authors:** Mustafa Mahmoud Aboulsaad, Olivier Donzel-Gargand, Rafael B. Araujo, Tomas Edvinsson

**Affiliations:** † Department of Materials Science and Engineering, Solid State Physics, 8097 Uppsala University, P.O. Box 35, 75103 Uppsala, Sweden; ‡ Department of Materials Science and Engineering, Solar Cell Technology, 8097 Uppsala University, P.O. Box 35, 75103 Uppsala, Sweden

## Abstract

Quantum confinement in low-dimensional semiconductors modifies not only electronic states but also the underlying lattice dynamics. In halide perovskite nanoplatelets, however, how confinement influences vibrational behavior alongside structural and optical properties remains an open question. Here, we synthesize CsPbBr_3_ nanoplatelets with atomically defined thicknesses from 2 to 5 monolayers and examine their structural, optical, and vibrational responses. Structural characterization combined with photoluminescence spectroscopy and first-principles calculations confirms reliable thickness control and structural stability across the series. Using polarization-resolved Raman spectroscopy, we observe gradual and systematic changes in the intensities of low-frequency lattice vibrational modes as the nanoplatelet thickness increases. First-principles calculations reveal that these trends arise from thickness-dependent changes in the spatial distribution of atomic displacements, with in-plane Pb–Br–Pb bending modes evolving more strongly than modes involving out-of-plane distortions. Similar behavior is found in iodine-based perovskite nanoplatelets, suggesting that the observed trends reflect a general feature of confined halide perovskites. Together, these results show that Raman intensity ratios, particularly in cross-polarized geometries, offer a physically grounded and nondestructive way to track thickness-dependent lattice dynamics in the 2–5 monolayer regime. More broadly, dimensional control provides a pathway to modulate vibrational dynamics and related energy relaxation processes, with implications for 2D materials at interfaces and in miniaturized devices.

## Graphical abstract

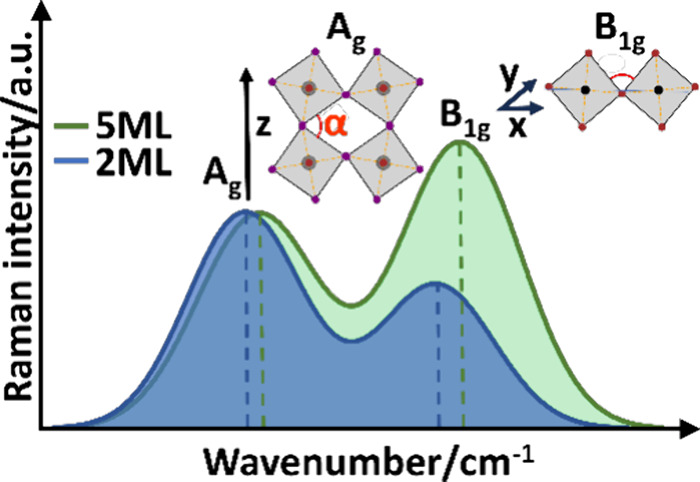

Two-dimensional (2D) halide perovskite nanoplatelets (NPLs) have emerged as transformative materials in optoelectronics, promising applications in light-emitting diodes (LEDs), lasers, and photodetectors. Their appeal lies in highly tunable electronic and optical properties engineered for specific device requirements. Early tuning approaches emphasized compositional engineering of the perovskite lattice, such as halide substitution or variation of the primary cation.^1^ More recently, attention has shifted to exploiting quantum confinement from reduced thickness since it alters the electronic structure by spatially localizing charge carriers, reshaping the density of states (DOS), exciton binding energy, and bandgap.^2^ A key manifestation of the confinement effect is the systematic blue shift in emission with decreasing thickness, reflecting reduced dimensionality.^3–5^ Thus, NPLs provide a versatile platform for both device applications and fundamental studies of size-dependent quantum phenomena.

Extensive research has examined confinement effects on electronic structure and photoluminescence (PL). Reports consistently show that reduced thickness modulates PL via quantum confinement and carrier-phonon coupling.^6–8^ For instance, and in connection to our work, Bohn et al. showed that such PL modulation can be utilized to identify the thickness of CsPbBr_3_ NPls through the peak position of PL emission.^9^ Yet, vibrational properties across different thicknesses remain less understood, with only limited studies available.^10–14^ In contrast to layered van der Waals materials, such as transition-metal dichalcogenides, inorganic halide perovskites are not intrinsically layered in their bulk form.^15^ As a result, reducing their thickness introduces not only confinement but also surface termination effects, structural relaxation, and possible phase competition, all of which can influence lattice vibrations.

Raman spectroscopy offers a powerful, nondestructive probe of lattice dynamics in low-dimensional materials. For instance, Raman has been indispensable in 2D materials such as graphene, where the G (∼1580 cm^–1^) and the overtone of the first-order forbidden D mode (∼1350 cm^–1^) appear as the 2D (∼2700 cm^–1^) band, enabling the ability to distinguish mono- to trilayers from multilayers and graphite.^16^ In nanoparticles, confinement and symmetry lowering relax the q≈0 selection rule, enabling off-center phonons to contribute. This generates thickness-dependent frequency shifts, line-shape asymmetries, and altered intensity ratios of symmetry-allowed channels, establishing Raman as a precise probe of confinement.^17–19^ Similar size-dependent Raman responses are also reported in oxides such as TiO_2_ and ZnO.^17–20^

Beyond confinement, Raman is highly sensitive to anisotropy and symmetry breaking,^21,22^ enabling layer-number identification and detection of subtle perturbations. Polarization-resolved approaches strengthen this capability: by isolating symmetry channels (co- vs cross-polarized geometries), one can quantify interlayer coupling^23^ and surface localization, and derive symmetry-resolved metrics, such as cross/copolarized ratios and line shape asymmetry parameter, that directly report on confinement and provide calibrated thickness readouts. In the last decades, this approach has been achieved for crystalline materials such as graphene and TMDs, mainly having hexagonal crystal structure with a small basis (2 atoms in the basis). Here, either intensity ratio changes (graphene) or line-shape/frequency shifts (TMDs) are utilized, while the situation for materials with a larger basis and their precise polarization changes as a function of 2D confinement have been elusive.

We synthesized CsPbBr_3_ nanoplatelets with well-defined thicknesses of two to five monolayers, together with nanocrystals (NCs) used as bulk-like references. Structural characterization by X-ray diffraction and electron microscopy confirms mixed orthorhombic–cubic frameworks and discrete layer counts across the nanoplatelet series. Optical absorption and photoluminescence measurements show the expected quantum-confinement-induced blue shift and systematic line width alteration with decreasing thickness, providing further confirmation of thickness control. Building on this foundation and emphasizing the reliable thickness assignment of the 2–5 monolayer samples, we performed polarization-resolved Raman spectroscopy to probe how lattice vibrations evolve under confinement. Despite the subtle nature of the spectral changes, clear mode-dependent trends with thickness are observed. In particular, vibrational modes dominated by in-plane Pb–Br–Pb bending exhibit a stronger thickness dependence than modes associated with out-of-plane distortions. First-principles calculations provide a microscopic interpretation of these trends, showing that in-plane modes increasingly localize within interior octahedral layers as thickness increases, whereas out-of-plane modes remain strongly influenced by surface termination and finite-size effects. To assess the generality of this framework, we extended our analysis to iodine-based perovskite nanoplatelets and observed the same thickness-dependent trends in Raman intensity ratios. We further validated these findings by examining the influence of postsynthetic treatments on the colloidal stability and vibrational properties of the nanoplatelets. Together, these results indicate that the proposed framework is broadly applicable across two-dimensional halide perovskite systems and, more generally, to other layered materials with related Raman-active vibrational modes.

Beyond offering insight into the lattice dynamics of confined halide perovskites, these results suggest that polarization-dependent Raman intensity ratios could, in future studies, be exploited as a nondestructive spectroscopic indicator of nanoplatelet thickness. Together, this work establishes a unified framework for understanding how dimensional confinement governs structural stability, optical response, and lattice dynamics in halide perovskite nanoplatelets.

CsPbBr_3_, like other perovskites, can adopt multiple crystal phases over a wide temperature range. At room temperature, both single-crystal and polycrystalline forms exhibit an orthorhombic phase; upon heating, single crystals transform to a tetragonal phase at 126 °C, whereas polycrystals transform to a cubic phase at 130 °C.^24,25^ In NCs, the phase behavior can deviate from that of the bulk. Under specific experimental conditions, CsPbBr_3_ NCs can exhibit different crystal structures at room temperature, transitioning to alternative phases within specific temperature windows.^26,27^ For example, NCs reported to have an orthorhombic phase at room temperature may convert to a cubic phase at 117 °C.^24^ In addition, a tetragonal phase in the *I*4/*mcm* space group has been observed in single crystals^28^ and in nanocrystal thin films over a broad temperature range (7 to 360 K).^29^

The exact crystal structure of nanosized CsPbBr_3_ has been the subject of extensive debate. Many XRD studies report cubic NCs, whereas others identify orthorhombic structures, as in the work of Cottingham and Brutchey,^30^ who employed high-resolution synchrotron X-ray pair distribution function (PDF) analyses on 6.5 and 12.5 nm NCs. Brennan et al. further investigated individual ∼ 10 nm NCs using high-resolution TEM, revealing a majority cubic and minority orthorhombic phase distribution.^31^ While differences at the individual NC level have been observed by electron microscopy,^32,33^ the prevailing consensus in recent literature is that the lowest-symmetry orthorhombic phase is typically the stable crystal structure for CsPbBr_3_ nanocrystals under standard conditions.^24,30,34^

Our X-ray diffraction (XRD) measurements reveal primary diffraction peaks across all samples, most notably at 15° and 31° in 2θ (Figure 1a). In principle, identifying the orthorhombic phase of CsPbBr_3_ should be straightforward, as this phase exhibits a much higher density of diffraction peaks compared to the cubic phase.^24^ However, in practice, Scherrer broadening smears together the closely spaced reflections, leading to broader peaks than those expected for the cubic phase. For bulk-like nanocrystals (NCs), the orthorhombic crystal structure can nevertheless be distinguished by additional weak diffraction features (labeled with stars in Figure 1b) that arise from the reduced lattice symmetry and are absent in the cubic phase.^24^ Further evidence comes from shoulders or asymmetric line shapes in the reflection profiles, particularly prominent in the 5 ML and bulk-like NC samples (Figure 1b,c).^33^ Similar distortions are also observed in CsPbBr_3_ nanoplatelets (NPLs), and prior studies have confirmed that such NPLs adopt an orthorhombic structure, reflecting a slight microscopic in-plane symmetry breaking.^35,36^ Variations in peak intensity ratios across different samples most likely originate from anisotropic orientation effects introduced during drop-casting.^33^ Collectively, these diffraction features and symmetry-related distortions provide strong evidence that the samples retain an orthorhombic crystal structure. Importantly, no diffraction peaks corresponding to Cs_4_PbBr_6_, a zero-dimensional phase associated with NPL instability, were detected in any of the samples.^37^

**Figure 1:**
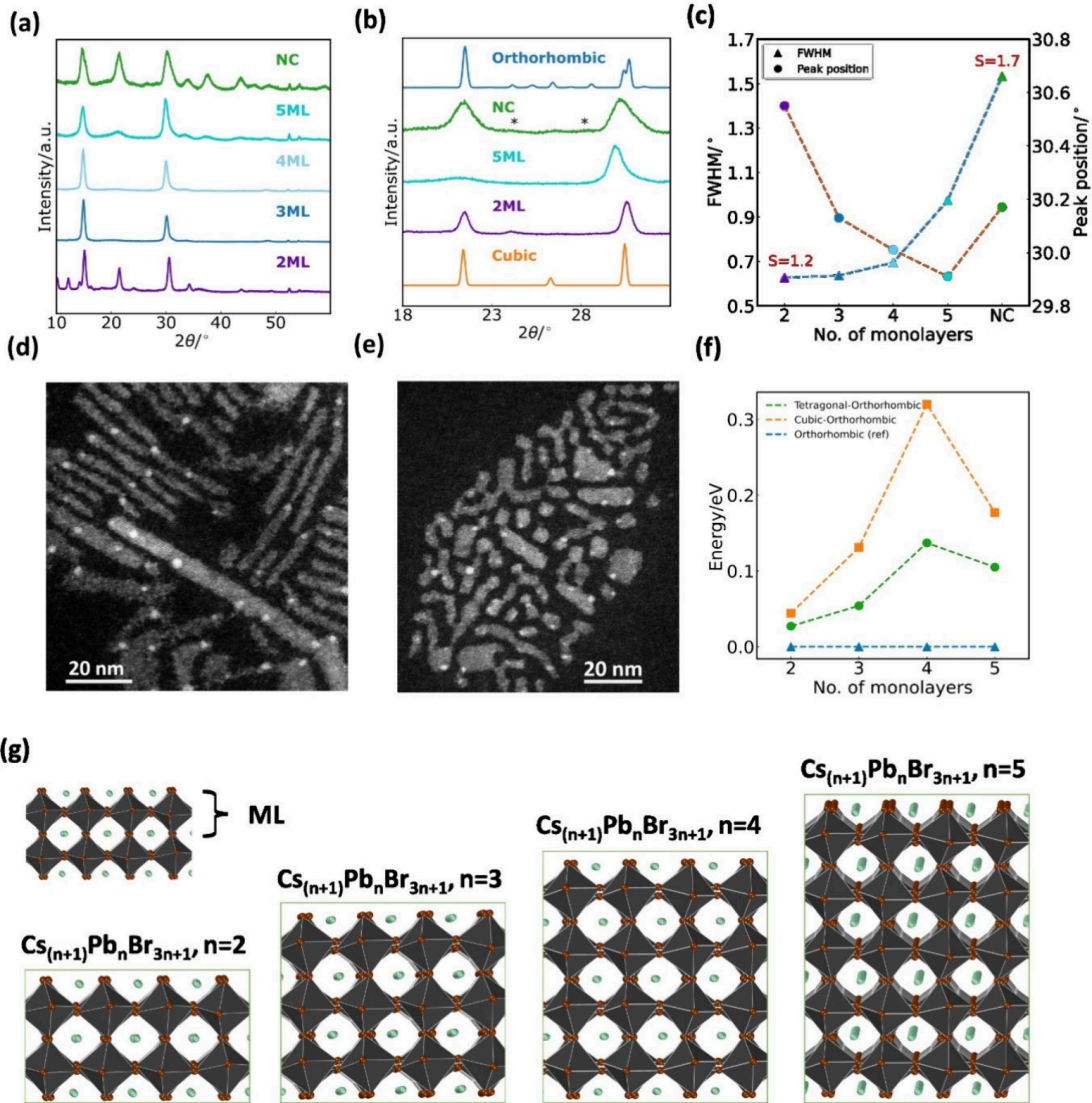
(a) GIXRD for the nanocrystal systems indicating the peak of interest at 31°. (b) Orthorhombic features of NCs compared with cubic and orthorhombic phases of CsPbBr_3_, as well as 2 and 5 MLs NPLs. (c) FWHM and peak shift analysis of the diffraction peak at 31°. S is the symmetrical parameter, where S = 1 indicates the highest symmetry. The full method for the calculation of the symmetry factor is in the Supporting Information. (d) and (e) STEM annular dark-field images showing the agglomerations of 3 and 5 MLs NPLs, respectively. (f) Relative total energies of CsPbBr_3_ NPls with 2–5 MLs, referenced to the orthorhombic phase. The cubic–orthorhombic (orange squares) and tetragonal–orthorhombic (blue circles). Here, the ground state case from the minima hopping was employed for all cases. (g) Orthorhombic structural models for NPLs calculations.

A distinct deviation was observed for the 2 ML sample (Figure 1a), which exhibited additional low-angle peaks at 10.1°, 12.2°, 14.2°, 16.3°, and 18.4° in 2θ. These reflections correspond to lamellar spacings of approximately 4.2 nm, characteristic of stacked NPLs in the solid state, thereby confirming that the NPL geometry is preserved without evidence of shape transformation.^38,39^ Another notable trend is the systematic shift of diffraction peaks to higher 2θ values as the number of monolayers decreases, which can be attributed to the increased surface energy of thinner NPLs due to their higher surface-to-volume ratio.^40–43^ At the same time, orthorhombic features, including the weak peaks marked with stars (Figure 1b), the full width at half-maximum of the 31° reflection (Figure 1c), and the asymmetric peak shoulders, become less pronounced as the number of monolayers decreases. This evolution is captured by the asymmetry parameter S, which increases upon thinning and indicates a gradual transition toward a more cubic-like character (Figure 1c). Nanocrystals exhibit stronger peak asymmetry than the 2-monolayer samples. A similar thickness-dependent structural evolution has previously been reported by Wang et al., who attributed this trend to changes in the Cs:Pb precursors ratio, where the number of monolayers increases with the increase of this ratio.^44^

First-principle calculations were employed to clarify the NPL most likely structures. Two-dimensional slab models were constructed from optimized bulk orthorhombic, cubic, and tetragonal phases of CsPbBr_3_ (for the cubic case, a supercell of 2 × 2 × 2 was employed to ensure that the small octahedral distortions are present). Slabs containing two to five atomic layers were generated and separated by a vacuum region of at least 20 Å along the out-of-plane direction to avoid interactions between periodic images. Slab terminations were chosen to eliminate dangling bonds and ensure structural stability. To identify low-energy configurations and account for competing polymorphs, all models were subjected to global structural optimization using the minima hopping approach. Unless otherwise specified, lattice parameters were kept fixed (more details can be found in the Supporting Information (SI)). For each thickness, the lowest-energy configuration was identified and used for comparison. The calculated energy differences between phases increase with the number of layers, indicating a progressive stabilization of the orthorhombic structure as thickness increases. This trend is illustrated in Figure 1f, which summarizes the relative energies, while representative lowest-energy orthorhombic structures are shown in Figure 1g. In contrast, the tetragonal phase remains energetically unfavorable across all thicknesses considered. For the two-monolayer case, the energy difference between the orthorhombic and cubic phases is only 0.027 eV, which is comparable to thermal energy at room temperature (kBT ≈ 0.026 eV). This small energy separation suggests that both phases may coexist at this thickness. As the number of layers increases, the orthorhombic phase becomes increasingly favored, confirming it as the dominant and more stable structure at larger thicknesses.

Figures 1d,e show TEM images for the formation of 3- and 5-MLs NPLs. Dark-field images reveal sparse, intensely bright nanodots that we assign to metallic Pb precipitates, consistent with electron-beam–induced halogen loss and radiolytic reduction of Pb^2+^ to Pb^0^ reported for lead-halide perovskites.^45^ 4D-STEM diffraction from the same regions shows mixed cubic and orthorhombic features, in line with crystallographic heterogeneity at the single-particle level.^31^ The material is quite sensitive to the electron beam, most of the lattice contrast disappearing after the first scan, as shown in the SI (Figure S2).^46^ Under continuous illumination, a few unidentified SAED rings weaken or vanish within seconds, giving evidence for beam-driven sensitivity; thus, TEM diffraction and high-resolution imaging should be interpreted with caution and acquired under low-dose conditions.^46^ More details about beam sensitivity and the EM diffraction analysis can be found in the SI (Figures S2–S8).

The PL spectra (Figure 2a) exhibit narrow peaks (fwhm: 12.7 nm for 2 ML to 21 nm for 5 ML) with emission shifting from 488 to 431 nm as the Cs/Pb ratio decreases, i.e., yielding thinner nanoplatelets. Under UV, this corresponds to a visible change from deep blue (2 ML) to cyan (5 ML), and to green emission at 512 nm (fwhm = 20 nm) for the biggest nanocrystals (NCs), consistent with weak quantum confinement in big NCs.^5,47^ Absorption spectra (Figure 2b) display sharp onsets and Stokes shifts of 4–23 nm. At lower Cs/Pb ratios, pronounced excitonic peaks and step-like continuum absorption, characteristic of 2D semiconductors, are observed, reflecting increased exciton binding energy (EB) and reduced exciton dissociation. These excitonic features weaken with increasing thickness and vanish in NCs.

**Figure 2:**
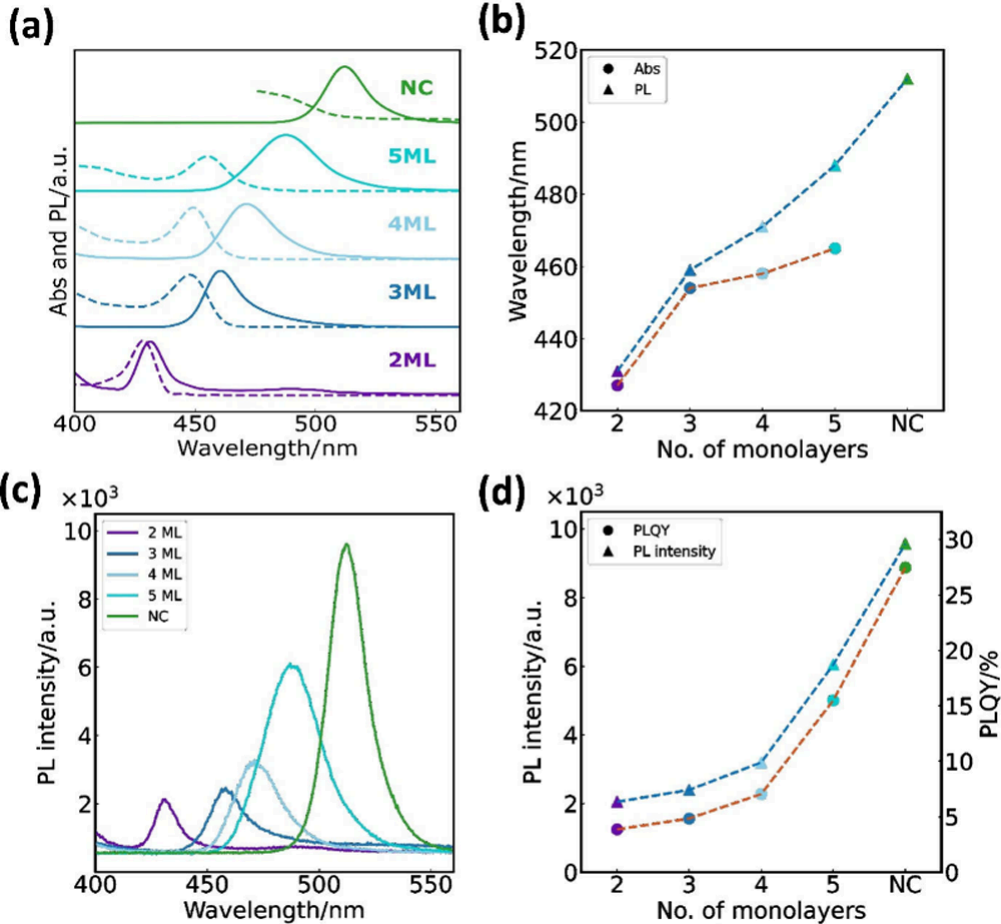
(a) Normalized PL (solid lines) and absorption (dashed lines) spectra of NPls films for varying NPl thickness. Spectra of weakly confined NCs are also shown for comparison, (b) Peak positions for the excitonic absorption/emission maxima for the determination of Stokes shift change with monolayer. The absorption maximum does not exist for bigger NCs due to the absence of an excitonic feature, (c) Non-normalized PL spectra showing the PL enhancement with the number of monolayers, and (d) PL and PLQY intensities.

The PL blue shift at reduced Cs/Pb ratios originates from quantum confinement, which raises the continuum onset energy (E_C_ = E_G_ + E_e_ + E_h_). Enhanced Coulomb interactions in confined dimensions increase EB, shifting EC upward. PL intensity rises exponentially from 2 MLs to 5 MLs (Figure 2c) due to more photoactive sites, while photoluminescence quantum yield (PLQY) remains low; 4% (2 ML), 5% (3 ML), versus 27% for NCs (Figure 2d), likely due to trap states and large surface-to-volume ratios. These energies and PLQYs compare well with those reported by Bohn et al.,^9^ while the lower PLQYs in our synthesis indicate slightly higher trap densities.

A comprehensive excitonic analysis of CsPbBr_3_ nanoplatelets based on the model Bethe–Salpeter equation is presented in a separate study by the authors^48^ and is therefore only briefly summarized here. In that work, calculations based on the orthorhombic phase reproduce the position of the first excitonic peak and capture the experimentally observed blue shift with decreasing thickness. The calculated exciton binding energy decreases only modestly with increasing thickness, from 0.26 eV for two monolayers to 0.21 eV for five monolayers, indicating that changes in the electronic band structure dominate the spectral shift. The consistency between these calculations and the photoluminescence measurements presented here supports the assigned thickness of the nanoplatelets across the two- to five-monolayer regime.

Owing to the confocal configuration, Raman spectra were acquired at the same spatial locations as the PL measurements. As a result, PL served as a predefined reference, ensuring that Raman measurements selectively probed nanoplatelets with a specific and well-defined number of monolayers. The Raman spectra of NPLs and NCs display fundamental bands centered at 47 cm^–1^, 73 cm^–1^, and 126 cm^–1^ (Figure 3a) for all samples. The two lower-frequency bands dominate in intensity, while the 126 cm^–1^ band is weaker. A clear enhancement of the 73 cm^–1^ band relative to the others is observed when progressing from 2 ML to the NC case, quantified through the intensity ratio of the 47 cm^–1^ and 73 cm^–1^ bands (Figure 3b). In 2 ML NPLs, the 47 cm^–1^ mode dominates, whereas in 3 ML the intensities are comparable. For thicker samples, the 73 cm^–1^ mode remains stronger than the 47 cm^–1^ mode. Additionally, a red shift of the 73 cm^–1^ mode is observed from 2 ML to the NC case, which is beyond the energy resolution of the instrument (0.6 cm^–1^) (Figure 3c), while the 47 cm^–1^ band shows only a weak blue shift, evident between 5 ML and the NCs.

**Figure 3:**
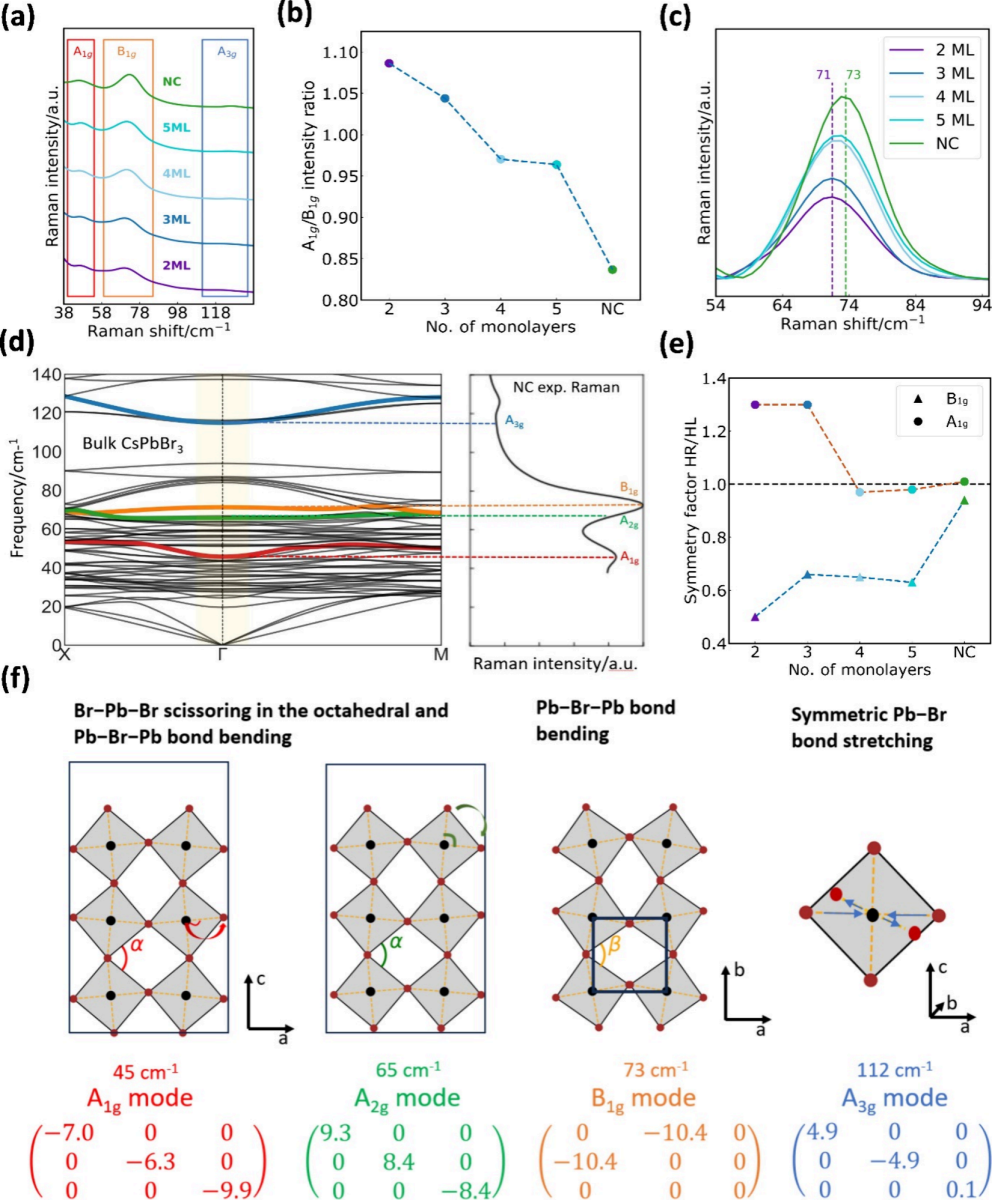
(a) Raman spectra for the 2 ML, 3 ML, 4 ML, 5 ML NPLs, and NCs. Here, emphasis is given to the observed three main peaks at 47 cm^–1^, 73 cm^–1^ and weak feature at around 126 cm^–1^. (b) Mode intensity ratio (A_1g_/B_1g_) for each NPLs and the NC. (c) Close look on the Raman spectra around mode B_1g_, showing a red shift of the mode frequency with decreased number of MLs. (d) Calculated phonon band structure of orthorhombic CsPbBr_3_, highlighting the Raman-active modes at the Γ point. (e) Symmetry factor of A_1g_ and B_1g_ modes and (f) Vibrational eigenmodes (A_1g_, A_2g_, A_3g_, and B_1g_) with atomic displacement patterns. The Raman tensor components for each mode are shown, emphasizing their symmetry and selection rules.

To further assess the robustness of the vibrational signatures against postsynthetic processing, we investigated the effect of different antisolvent treatments on 3 ML nanoplatelets using Raman and photoluminescence (PL) spectroscopy (Figure S9). Raman and PL spectra were compared for pristine 3 ML NPLs and for NPLs subjected to washing with different antisolvents. Purification using hexane combined with centrifugation preserved the characteristic spectral features of the 3 ML nanoplatelets, while effectively removing contributions from larger platelets, as reflected by subtle changes in spectral intensity and line width. In contrast, treatment with more polar antisolvents such as ethyl acetate or methyl acetate led to noticeable modifications in both Raman and PL spectra, consistent with aggregation or growth of thicker nanoplatelets. These observations highlight the strong sensitivity of both Raman and PL spectroscopy to postsynthetic processing and confirm their suitability for monitoring nanoplatelet integrity and size evolution.

Separately, to evaluate the generality of the thickness-dependent vibrational behavior across halide compositions, we extended our Raman analysis to iodine-based perovskite nanoplatelets (Figure S10). While the detailed thickness-dependent Raman analysis is established for bromide-based nanoplatelets, the iodide system is introduced here to probe the generality of the symmetry-driven trends across halide composition. The iodide data therefore serve as a qualitative validation of the underlying mechanism rather than as a full quantitative benchmark. Despite the different halide chemistry, the relative contributions of in-plane and out-of-plane vibrational modes evolve systematically with increasing thickness from 3 to 5 monolayers, closely resembling the behavior observed in bromide-based systems. This consistency indicates that the underlying vibrational response is governed by symmetry and confinement effects rather than by specific halide chemistry, pointing to a generic mechanism in layered halide perovskites. More information about the synthesis of CsPbI_3_ and the characterization parameters is in the SI.

To assign the experimentally observed vibrational modes, Raman spectra and vibrational properties were computed for both the bulk phase and nanoplatelets (slab models), focusing on the orthorhombic structure, which was found to be the most energetically stable phase. Raman calculations were performed within the Placzek approximation,^49^ where the mode intensities are given by

$$I_{i}\propto \left(\frac{n\left(w_{i},T\right)+1)}{w_{i}}\right){A_{i}}^{2}$$ 1

where

$$A_{i}=e_{\mathrm{out}}^{T}R_{i}e_{\mathrm{in}}$$ 2

and

$$n\left(w,T\right)=\frac{1}{\exp^{\left(\hbar w/k_{B}T\right)}-1}$$ 3

Calculations were carried out at T = 300 K. Polarization unit vectors were defined as xx (*e*_*in*_= (1,0,0), *e*_*out*_ = (1,0,0)), xy (*e*_*in*_ = (1,0,0), *e*_*out*_ = (0,1,0)), and yy (*e*_*in*_ = (0,1,0), *e*_*out*_ = (0,1,0)). Polarizability tensors *R*_*i*_ were obtained by applying finite differences along each vibrational mode (atoms are displaced by +0.005 Å and – 0.005 Å). This framework allows quantitative comparison between calculated Raman tensors and experimental polarization-resolved spectra (more details on the vibrational modes and Raman intensities are in the SI). Calculations for the bulk orthorhombic phase showed no imaginary phonon frequencies, confirming stability (Figure 3d). The zone-center optical modes decompose as Γ_optic_ = 7A_g_ + 8A_u_ + 5B_1g_ + 9B_1u_ + 7B_2g_ + 7B_2u_ + 5B_3g_ + 9B_3u_, of which 7A_g_ + 5B_1g_ + 7B_2g_ + 5B_3g_ are Raman active. From these, four dominant modes were predicted at 45, 65, 73, and 112 cm^–1^ (Figure 3d and Figure S13). Experimentally, NCs showed bands at 47, 73, and 126 cm^–1^, in good agreement with theory, though the 65 cm^–1^ mode overlaps with the 73 cm^–1^ feature, appearing as a broadened band at room temperature. This correspondence indicates consistent vibrational behavior between NPLs and the performed calculations. Analysis of polarizability tensors assigns the 45 cm^–1^ mode to an A_g_ (indexed as A_1g_ for clarity) symmetry mode with Br–Pb–Br scissoring and Pb–Br–Pb bending along z (α angle), the 65 cm^–1^ A_g_ (indexed as A_2g_) mode to a related scissoring and bending with distinct distortion, the 73 cm^–1^ B_1g_ mode to Pb–Br–Pb bending within the xy plane, and the 112 cm^–1^ A_g_ (indexed as A_3g_) mode to symmetric Pb–Br stretching (Figure 3f).

To capture thickness effects, Raman spectra were also computed for the slab models built with the orthorhombic block unit. In a backscattering geometry, the parallel polarization channels (xx, yy) are dominated by contributions from A_1g_ modes, while the crossed polarization channel (xy) selectively probes Raman tensor components of B_1g_ symmetry. Possible in-plane rotational disorder of the nanoplatelets (domains) may partially relax these selection rules, leading to weak symmetry mixing; however, the dominant polarization dependence remains governed by the underlying orthorhombic Raman tensors. As thickness increases from 2 to 5 ML, overall intensity grows due to layer accumulation. Experimentally, peaks at 47 cm^–1^ and 71–73 cm^–1^ align well with the theoretical A_1g_ (xx) and B_1g_ (xy) modes at 45 cm^–1^ and 73 cm^–1^ (Figure 3a, 3f), with the strongest enhancement in intensity for B_1g_ mode as the thickness grows (Figure 3c).

A quantitative comparison of the polarization-dependent Raman response is provided by a bar plot of the absolute peak-difference metric

$$\left|△I\right|=\left|I_{\mathrm{xx}}^{A1g}-I_{\mathrm{xy}}^{B1g}\right|$$ 4

referenced to the low-frequency band in the xx configuration (Figure 4b, left). This metric directly measures the relative weight of totally symmetric (A_1g_) and crossed-polarization (B_1g_) contributions as a function of nanoplatelet thickness. The xx-polarized A_1g_ modes do not exhibit a systematic increase with thickness: in particular, the 5-monolayer (ML) nanoplatelets show differences comparable to—or even smaller than—those of the 4-ML sample. In contrast, the B_1g_-related differences extracted from the xy channel increase monotonically from 2 to 5 ML, indicating a progressive enhancement of the Raman intensities as a product of the increasing thickness. To disentangle intrinsic thickness effects from trivial scaling with the number of layers, each spectrum is further normalized by the corresponding layer count (Figure 4b, right). Under this normalization, the per-layer contribution of the xx-polarized A_1g_ modes is slightly reduced with increasing thickness, whereas the xy-polarized signal continues to grow monotonically across the entire 2–5 ML range. Finally, the weak and broad experimental feature observed near 125 cm^–1^ in nanocrystals, progressively suppressed upon dimensional reduction to nanoplatelets, is consistent with the low-intensity A_3g_ mode predicted by theory at approximately 120 cm^–1^.

**Figure 4:**
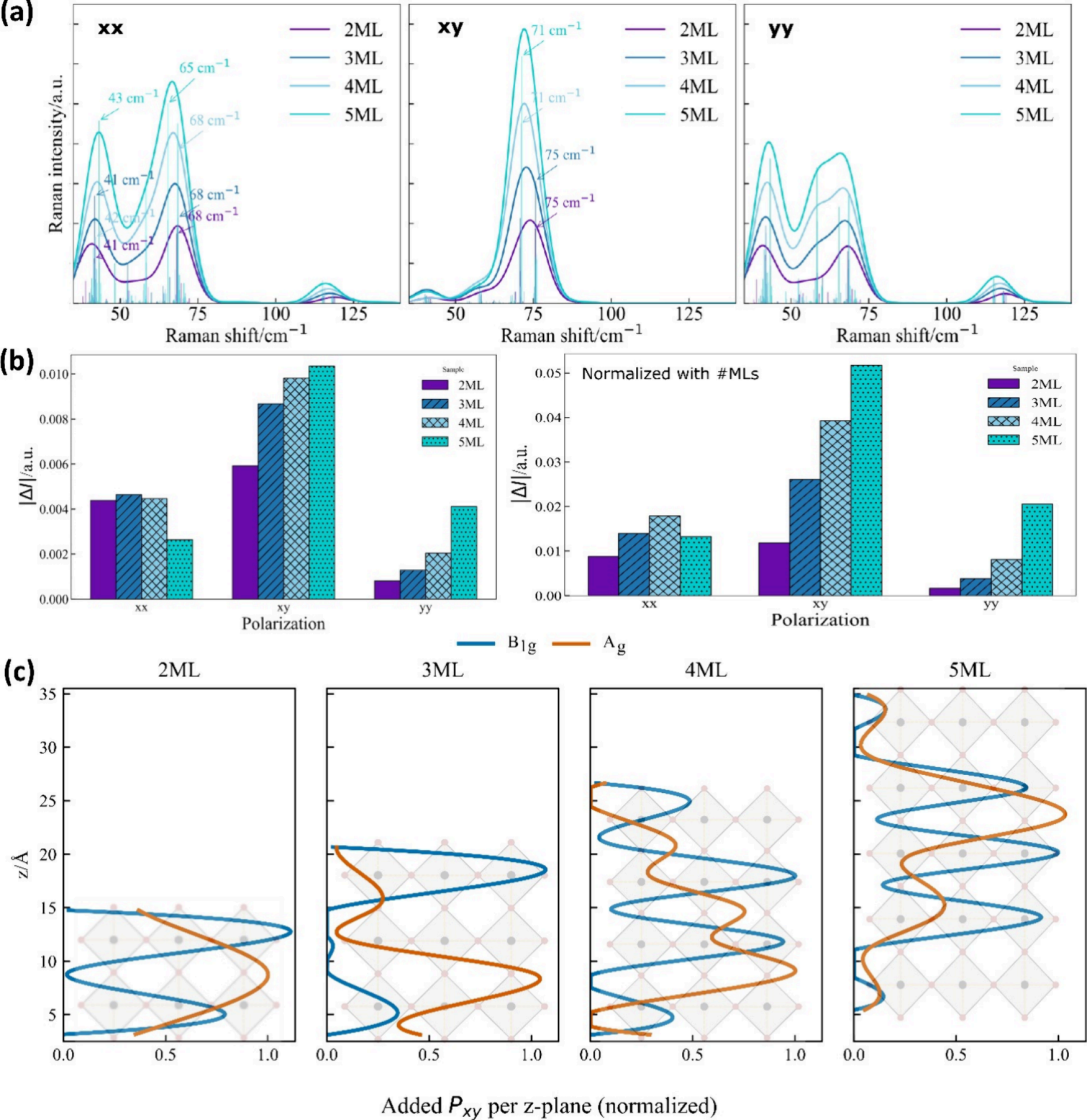
(a) Polarization-resolved Raman spectra (xx, xy, yy) for 2–5 MLs, highlighting systematic intensity changes of the main phonon bands. (b) Quantitative analysis of thickness-dependent Raman intensity variations shows that B_1g_ modes grow faster with thickness, as calculated (left) and normalized with the number of MLs (right). (c) DFT-calculated plane-resolved polarizability contributions P_*xy*_ for B_1g_ and A_1g_ modes across thicknesses, revealing that B_1g_ displacements are confined within interior octahedra, while A_1g_ modes involve surface-connected distortions (parameter α), explaining their slower intensity growth with layer number.

The intensity ratio between selected Raman modes can be quantitatively explained using the phonon confinement model, first proposed by Richter et al.^50^ and Campbell and Fauchet.^51^ The approach predicts how a reduction in the crystal dimensions down to the nanoscale relaxes the first-order Raman selection rule, which in bulk restricts scattering to zone-center phonons (q = 0). In quantum dots, as shown and further refined for ZnO in comparison with first-principle calculations by Edvinsson and co-workers,^20^ the finite lattice size confines the phonon wave function, broadening its momentum distribution and enabling contributions from q ≠ 0 states. An equation for the intensity and shape of the phonon modes via the relaxation of the q = 0 condition can be formulated as^20,51^

$$I\left(\omega ,d\right)=A\int \frac{\exp\left(-\frac{q^{2}d^{2}}{16\pi^{2}}\right)}{{\left(\omega \left(0\right)-\Delta \omega \cdot \sin^{2}\left(\frac{qa}{4}\right)-\omega \left(q\right)\right)}^{2}+{\left(\frac{\Gamma_{0}}{2}\right)}^{2}}d^{3}q$$ 5

where *I* is Raman intensity, ω vibrational frequency, *d* particle diameter, *A* an intensity prefactor depending on the thickness and cross-section of the polarizability of the bonds in the sample, *ω­(0)* zone center phonon frequency, *Δω* the difference between the zone center and the zone boundary frequency, *ω­(q)* phonon dispersion, Γ_0_ natural full width at half-maximum, *q* wave vector, and *a* the lattice parameter in the direction of the vibrational mode. The integration is performed over the whole Brillouin zone. For a 2D system, the phonon dispersion *ω­(q)* has a functional form of $\omega^{2}\left(k_{x}k_{y}\right)=\frac{k_{x}+k_{y}}{m}$ leading to a reduced form of the expression with integration over *d* ^2^ *q*, neglecting *k*_*z*_. In our case, however, as we start from a 2 ML system with successively larger extensions to 3, 4, and 5 MLs in the *k*_*z*_ direction, the effects from extension in the *z*-direction are vital to describe the successive change, where a 3D description is required with varied restrictions of material dimensions in the *z*-direction.

Although eq 1 represents an idealized situation, it provides a starting point to analyze general trends. Since the phonon frequency *ω­(q)* varies across the Brillouin zone and considering the relaxation of the *q = 0* selection rule for quantum confined materials, one expects a decrease of the phase coherence and a dimensionally induced peak broadening and frequency shifts determined by the dispersion. Negative dispersion (Δω < 0) yields asymmetric, red-shifted tails, while positive dispersion (Δω > 0) gives blue-shifted tails. In bulk CsPbBr_3_, the B_1__g_ branch shows a slight negative dispersion near the Brillouin-zone center (Figure 3d), so eq 5 predicts a confinement-induced red shift toward lower wavenumbers. Indeed, 4 ML, 5 ML, and NC samples exhibit B_1__g_ broadening toward lower wavenumbers for 2 MLs, with a small shift (2 cm^–1^), indicating a small dispersion of the phonon bands as the q = 0 selection rule is relaxed. However, in 2 and 3 ML samples, the B_1__g_ mode instead shows asymmetric broadening slightly toward higher wavenumbers (Figure 3c). Our DFT calculations reproduce this anomaly (Figure 4a): in ultrathin slabs, the phononic wavevector localizes at the surfaces (Figure S16), shifting the B_1__g_ peak upward. The main B_1__g_ peaks appear at 73 cm^–1^ for NC, 71.3 and 71.0 cm^–1^ for 5 and 4 ML, but shift to 75.6 cm^–1^ for 3 and 2 ML for the theoretical peaks. Thus, while the model links dispersion sign to asymmetry, surface contributions dominate at low thickness. This is captured experimentally by the symmetry factor S: values >1 reflect higher-wavenumber tails in 2–3 MLs, while values <1 indicate lower-wavenumber broadening in 4–5 MLs and NCs (Figure 3e).

The A_1g_ mode behaves differently. In bulk CsPbBr_3_ it has positive dispersion (Figure 3d), where eq 5 predicts a blue shift under confinement. Yet, our Raman spectra reveal no frequency shift, only consistent red-tail broadening with decreasing thickness, also reflected in S (Figure 3e). This departure from the confinement model resembles TiO_2_ NCs, where the E_g_ mode follows the expected behavior from the analytic phonon confinement model, but the A_1g_ mode does not.^17^ Similar failures in rutile TiO_2_ were attributed to nonstoichiometry and dielectric effects in surface-dominated structures. Such effects are likely here as well, since ultrathin CsPbBr_3_ NPLs have large surface fractions where disorder and ligand interactions dominate, obscuring the confinement trend for A_1g_.

The distinct polarization-dependent behaviors can instead be understood directly from the phonon displacement patterns. To quantify how each vibrational mode is distributed across the nanoplatelet thickness, we analyze the phonon eigenvectors by projecting their atomic displacements onto the in-plane directions and summing the squared components, ∑(*e*_*x*_^2^ + *e*_*y*_^2^), layer by layer along the out-of-plane (z) direction. This procedure defines a layer-resolved in-plane displacement profile, *P*_*xy*_, which captures how strongly each mode involves in-plane atomic motion as a function of depth within the slab (Figure 4c).

Across all thicknesses, the B_1__g_ modes are predominantly localized within the interior PbBr_6_ layers and are characterized by in-plane (xy) atomic displacements, whereas the A_1g_ modes exhibit a stronger contribution from surface layers and involve coupling between layers through out-of-plane distortions, captured by the Pb–Br–Pb angle α along the z direction. This contrast offers a clear spatial interpretation of vibrational behavior. In the bulk material, B_1g_ modes stem from the antisymmetric in-plane bending of the octahedra, whereas A_1g_ modes combine in-plane scissoring with out-of-plane coupling.

As the thickness decreases, these modes respond differently to the structural boundaries:

- A_1g_ modes are heavily influenced by surface termination, which disrupts their out-of-plane coupling along the *z*-axis.
- B_1g_ modes escape this surface effect, remaining largely confined to the interior of the nanoplatelets.

Because of this distinction, the Raman intensity of the B_1g_ modes increases much more sharply as thickness grows. Consequently, the cross-polarized xy channel, which is selectively sensitive to B_1g_ symmetry, exhibits the most pronounced thickness dependence. The calculated P_*xy*_ metrics, therefore, provide a direct explanation for the observed intensity ratios. We note that surface chemistry and local disorders are inherently intertwined with vibrational symmetry in ultrathin nanoplatelets. Ligand binding, surface reconstruction, and finite-size termination primarily affect modes that involve out-of-plane distortions and surface-connected bonds. In this sense, surface disorder does not represent an alternative explanation to the observed symmetry-dependent trends but rather reinforces them: A_1g_ modes, which couple to *z*-direction distortions, are intrinsically more susceptible to surface effects, whereas B_1__g_ modes remain protected due to their predominantly in-plane, interior-localized character.

In the theoretical modeling, organic ligands were not explicitly included, and the nanoplatelets were treated as ideal inorganic slabs. This approximation is justified because the primary objective of the calculations is to resolve the intrinsic vibrational symmetry, phonon localization, and layer-resolved displacement patterns of the perovskite lattice. Organic ligands mainly influence surface passivation, electrostatic screening, and disorder at the outermost layers, effects that predominantly impact modes involving out-of-plane or surface-connected distortions, as previously mentioned. As shown by the experimental–theoretical comparison, the symmetry-dependent trends, particularly the thickness evolution of the in-plane B_1__g_ modes, are well captured without explicit ligand treatment, indicating that the key mechanisms are governed by the inorganic framework. Importantly, neglecting ligands is therefore expected to affect absolute frequencies or line widths of surface-sensitive modes, but not the qualitative symmetry contrast or the relative localization trends that underpin the proposed Raman metrology.

Ultrathin 2 ML films can behave as relatively well-ordered superlattice-like assemblies with a preferred orientation, which may favor specific scattering directions. Several works report superlattice formation in ultrathin perovskite NPLs.^4,40,41^ This is consistent with multiple GIXRD reflections corresponding to lamellar spacings, characteristic of stacked NPLs.^33^ As the thickness increases, the emergence of twinned or rotated domains and increasing azimuthal disorder could enhance scattering channels that are active in the xy geometry. Within this picture, the observed intensity evolution may reflect a combination of intrinsic effects related to symmetry and interlayer coupling, together with extrinsic contributions associated with domain structure that preferentially enhance cross-polarized Raman response.

Thickness readout in CsPbBr_3_ nanoplatelets is governed by symmetry-dependent intensity trends, with frequency shifts leaving only a subtle signature. In graphene, the number of layers is determined from the evolution of the 2D band, which splits and changes shape with thickness.^52^ In MoS_2_ and related transition-metal dichalcogenides (TMDCs), the thickness dependence is tracked through the frequency separation between the microscopic in-plane E_2__g_ and out-of-plane A_1__g_ modes, which increases systematically with interlayer coupling.^53^ Both systems rely primarily on frequency shifts as the structural marker of dimensionality. In CsPbBr_3_ nanoplatelets, however, the Raman bands shift only weakly with thickness, and the decisive fingerprint is the symmetry-selective Raman intensity evolution. Consequently, while TMDCs exploit frequency shifts as thickness probes, halide perovskite nanoplatelets exhibit a distinct symmetry- and localization-driven Raman mechanism to explain the relative intensity ratio with thickness. In graphene, with two atoms in the basis, intensity ratios between the graphite peak (G, sp^2^) and the first overtone (2D) of the absent diamond peak (D, sp^3^) are utilized as monolayer metrology for 1–3 MLs,^54^ while determinations of MLs beyond 3 are challenging. Here, we show that one can go beyond this limit via analysis of the direction-dependent polarizabilities in low-dimensional materials to enable ML metrology for a higher number of MLs also in more complex structures, such as orthorhombic halide perovskites.

In this work, we synthesized CsPbBr_3_ nanoplatelets with atomically controlled thicknesses from 2 to 5 monolayers and systematically investigated their structural, optical, and vibrational properties using structural characterization, photoluminescence spectroscopy, polarization-resolved Raman spectroscopy, and first-principles calculations. We established a reliable, nondestructive approach to track thickness-dependent lattice dynamics and demonstrated that vibrational dynamics can be deliberately tuned through dimensional confinement. Building on this well-established structural foundation, polarization-resolved Raman spectroscopy and first-principles calculations reveal systematic thickness-dependent trends in the vibrational response that originate from the spatial localization of phonon modes across the nanoplatelet thickness. In particular, modes dominated by in-plane Pb–Br–Pb bending evolve differently from modes involving out-of-plane distortions, reflecting the distinct roles of interior and surface layers in confined structures. Extending the analysis to iodine-based nanoplatelets reveals similar behavior, indicating that these trends are not material-specific but reflect a general feature of confined halide perovskites. Beyond providing insight into phonon confinement, our results show that direction- and polarization-dependent Raman intensity ratios offer a physically motivated and nondestructive route to track thickness-dependent lattice dynamics over the 2–5 monolayer regime, even in structurally complex perovskite systems. Together, this work highlights the importance of considering lattice dynamics alongside electronic effects when describing confinement in low-dimensional optoelectronic materials.

## Methods

The preparation of CsPbBr_3_ nanoplatelets followed the approach by Bohn et al.^9^ with some modifications.

### Materials

Cs_2_CO_3_ (ceasium carbonate, 99\%), PbBr_2_ (lead­(II) bromide, 98\%), oleic acid (technical grade 90\%), oleylamine (technical grade 70\%), toluene (for HPLC, 99.9\%), acetone (for HPLC, 99.9\%), hexane (for HPLC, 97.0\%, GC) and were purchased from Sigma-Aldrich.

### Preparation of Precursors

Prior to precursor preparation, both oleic acid and oleylamine were preheated to 110 °C and subsequently filtered through a 0.2 μm syringe filter to remove any precipitated impurities. The cesium oleate (Cs-oleate) precursor was synthesized by dissolving 0.1 mmol of Cs_2_CO_3_ powder in 10 mL of oleic acid at 100 °C under continuous stirring until complete dissolution. The PbBr_2_ precursor solution was prepared separately by dissolving 0.1 mmol of PbBr_2_ along with 100 μL each of oleylamine and oleic acid in 10 mL of toluene at 100 °C. The coordinating ligands (oleic acid and oleylamine) facilitate the dissolution of PbBr_2_ in the nonpolar toluene medium. Both precursor solutions were subsequently transferred to and stored in a nitrogen-filled glovebox for further use.

### Synthesis of CsPbBr3 NPLs

All synthesis steps were conducted at room temperature in a standard fume hood. Control over the nanoplatelet (NPL) thickness was achieved by adjusting the volume ratios of the two precursors and the antisolvent employed during precipitation. In a typical procedure, varying amounts of the Cs-oleate precursor were rapidly injected into the PbBr_2_–ligand precursor solution under vigorous stirring to ensure homogeneous nucleation and complete crystallization of the NPLs. After 5 s, acetone was added in specific volumes as an antisolvent to promote NPL precipitation from the toluene phase. Following 1 min of stirring, the mixture was centrifuged at 4000 rpm for 3 min, and the resulting precipitate was redispersed in hexane to yield a stock solution with a concentration of 15 mg/mL.

At this stage, the solution was suitable for characterization; however, to improve colloidal stability and eliminate larger crystalline aggregates, a second centrifugation step was performed at 3000 rpm for 3 min without adding antisolvent. The supernatant was then carefully extracted using a syringe to minimize disturbance. This additional washing step ensures the removal of excess fatty solvents and bigger nanocrystals. Accurate determination of the final concentration was achieved through multiple weight measurements of the centrifuge tubes. The resulting stock solution was subsequently diluted to a working concentration of 5 mg/mL, which was used throughout the remainder of the study unless stated otherwise.

The optimized precursor volume ratios for obtaining NPLs with 2, 3, 4, and 5 monolayers (MLs) were as follows (Cs-oleate/PbBr_2_–ligand/acetone in μL/mL/mL): 150/3/2 for 2 MLs, 150/1.5/2 for 3 MLs, 150/1.2/2 for 4 MLs, and 200/1/2 for 5 MLs. For the 5 ML NPLs, an additional 0.2 mL of acetone was premixed with the PbBr_2_–ligand precursor prior to Cs-oleate injection to suppress the formation of bulk crystals during nucleation. For the bigger NCs, the ratios were 1000/1/4, while an additional 1 mL of acetone was added to the PbBr_2_-ligand precursor prior to the Cs-oleate injection.

### Characterization

All characterization measurements, unless otherwise specified, were performed at room temperature on drop-cast films prepared from NPL dispersions in hexane, deposited onto glass or gold substrates. Photoluminescence (PL) enhancement and PL quantum yield (PLQY) measurements were conducted in solution at matched concentrations to ensure consistency.

PL spectra were acquired using a Renishaw inVia confocal PL–Raman system equipped with a 405 nm excitation laser (fwhm ≈ 1 nm). UV–Vis absorption spectra were collected using an Agilent Cary 5000 spectrophotometer equipped with halogen and deuterium lamps, from films deposited on glass substrates. Raman spectroscopy was carried out on drop-cast films on gold-coated glass substrates using the Renishaw inVia confocal system with a 785 nm excitation source and spectral resolution of 0.6 cm^–1^. A 50× objective lens was employed, and the laser power was set to 83 mW, corresponding to a power density of approximately 5.3 × 10^7^ mW/mm^2^, based on a 1.4 μm laser spot size. This power density was the maximum threshold that could be applied without inducing degradation of the perovskite NPLs. Raman spectra were collected in both extended and static modes with an acquisition time of 10 s per scan, and a total of three accumulations per sample.

Scanning Transmission Electron Microscopy (STEM) was performed on fresh NPLs samples prepared by drop-casting hexane-dispersed NPLs (1 mg/mL) onto holey-carbon Cu grids (Ted Pella, 200 mesh). Samples were prepared within a few hours before analysis, air-dried at room temperature. STEM imaging was conducted using a Cs-probe corrected FEI Titan Themis 200 microscope equipped with a Schottky field emission gun and operated at an accelerating voltage of 200 kV. Beam current was maintained between 20 and 70 pA in STEM mode.

Grazing incidence X-ray diffraction (GIXRD) measurements were carried out on NPL films drop-cast on SiO_*x*_/Si substrates using a Malvern PANalytical Empyrean system equipped with a copper X-ray source. The incidence angle was fixed at 0.5° for all samples to minimize background scattering from the substrate.

### Density Functional Theory Calculations

Density Functional Theory (DFT) calculations were carried out utilizing the Projected Augmented Wave (PAW) approach within the Vienna Ab initio Simulation Package (VASP).^55,56^ The generalized gradient approximation with the Perdew, Burke, and Ernzerhof (PBE) parametrization to address the exchange and correlation terms within the Kohn–Sham Hamiltonian^57^ was employed (unless specified) with the addition of spin–orbit coupling (SOC). Plane waves were expanded to a cutoff of 400 eV (unless specified), and the Brillouin zone was sampled with different grids depending on the structural lattice (3 × 3 × 1 for the orthorhombic and 2 × 2 × 1 for cubic distorted slabs and tetragonal).

### Vibrational Modes and Raman Activities

To examine the phonon modes and frequencies at the Γ point, density functional perturbation theory (DFPT) was employed. During this phase, the employed structure underwent optimization with a force convergence target of 0.001 eV/Å and an energy convergence goal of 10^–8^ eV. Phonon band structure and density of states were computed using Phononpy.^58^

Raman spectra were calculated using polarizability derivatives within the nonresonant Placzek approximation—a well-established approach in ab initio Raman modeling.^59,60^ Mode amplitudes were obtained by projecting the Raman tensor onto the incident and detected polarization vectors, with intensities weighted by thermal occupation and phonon frequency at 300 K. Polarization configurations (xx, xy, yy) were defined to isolate symmetry-allowed scattering channels. The resulting discrete lines were broadened using Gaussian functions to mimic instrumental line widths, producing continuous spectra suitable for comparison with experiment. Full methodological details and convergence tests are given in the Supporting Information.

## Supplementary Material



## Data Availability

The VASP code is licensed software available from https://www.vasp.at/.

## Notes

Additional details on the theoretical background, structures used in the DFT calculations, polarizability tensors for the Raman calculations, the Raman and PL of the post-treatment approach, the Raman spectra of the Iodide system, the TEM images and electron diffraction analysis, and the methods and codes for the calculation of the symmetrical parameter used in X-ray diffraction and Raman sections (PDF)
