# Supplementary material for: Symmetry-Driven Phonon Confinement in 2D Halide Perovskites
Source: J Phys Chem Lett. 2026 Jun 14;17(25):7008–19. doi: 10.1021/acs.jpclett.6c01515 (PMC13312441; doi:10.1021/acs.jpclett.6c01515)
Supplement: Supplementary file 1 [file jz6c01515_si_001.pdf]

Supporting Information for

Symmetry-driven phonon confinement in 2D halide perovskites

Mustafa Mahmoud Aboulsaad[a], Olivier Donzel-Gargand[b], Rafael B. Araujo[a],, Tomas Edvinsson[a],**

a Department of Materials Science and Engineering, Solid State Physics, Uppsala University, Box 35, 75103 Uppsala, Sweden

b Department of Materials Science and Engineering, Solar Cell Technology, Uppsala University, Box 35, 75103 Uppsala, Sweden

** Corresponding authors: rafael.araujo@angstrom.uu.se, tomas.edvinsson@angstrom.uu.se*

Table of Contents

Table of Figures 3

TEM 4

- Sample overview 4
- Electron beam sensitivity - 3ML case 4
- TEM diffraction analysis: 4D-STEM nanoprobe diffraction 6

Raman and PL 11

- Chemical-treatment of CsPbBr3 nanocrystals 11
- Symmetrical parameters calculations 12
- Code for the calculation of HL, HR, FWHM, skewness, and symmetry factor 13

Computational method 15

- Structural Considerations and Computational method 15
- Raman Calculations 15

Bibliography 20

Table of Figures

Figure S1: STEM annular dark field of the 2ML, 3ML and 5 ML samples 4
Figure S2: Electron sensitivity in the case of 3 ML NPLs.. 4
Figure S3: STEM annular dark field of the 3 ML NPLs sample after a few scans 5
Figure S4: Nano-diffraction 4D-STEM map of 3ML NPLs 6
Figure S5: STEM nano-diffraction extracted from 4D-STEM dataset of the 2ML NPLs 7
Figure S6: STEM nano-diffraction extracted from 4D-STEM dataset of the 3ML NPLs 8
Figure S7: Two typical STEM nano-diffraction extracted from 4D-STEM dataset of the 3ML NPLs 9
Figure S8: STEM nano-diffraction extracted from 4D-STEM dataset of the 5ML NPLs 10
Figure S9: (a) Raman and (b) PL spectra for a pristine and post-treated 3 MLs NPLs 11
Figure S10: Raman spectrum of 3-5 MLs CsPbI3 12
Figure S11: Schematic plot illustrating the general peak profile of the peak symmetry analysis......... 13
Figure S12: Atomistic models of Cs(n+1)Pb□Br$_3$□$_{+1}$ nanoplatelets with different layer thicknesses. ... 17
Figure S13: Polarization-resolved Raman spectra of bulk orthorhombic $CsPbBr_3$ 17
Figure S14: Calculated Raman spectra of the 2ML $CsPbBr_3$ nanoplatelet with different plane-wave cutoff energies. 18
Figure S15: Calculated Raman spectra of the 2ML $CsPbBr_3$ nanoplatelet using different Brillouin-zone sampling grids. 18
Figure S16: Thickness-dependent vibrational eigenmodes of $CsPbBr_3$ nanoplatelets 19

Table 1: Optimized lattice parameters of bulk $CsPbBr_3$ phases used for constructing nanoplatelet models. 19

TEM

Sample overview

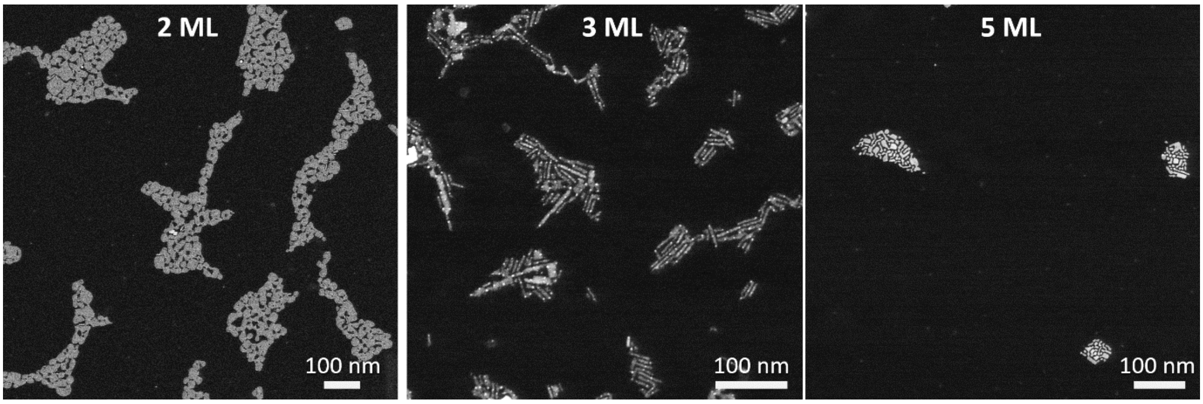

Figure S1: STEM annular dark field of the 2ML, 3ML and 5 ML samples after drop cast on a lacey carbon grid.

Electron beam sensitivity - 3ML case

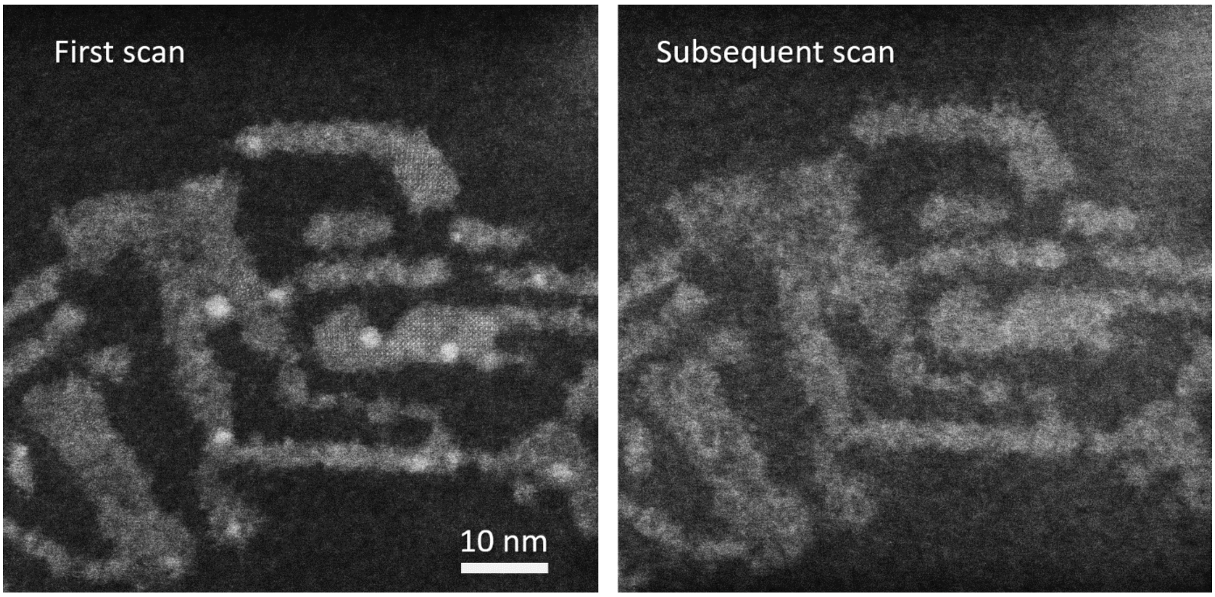

Figure S2: Electron sensitivity in the case of 3 ML NPLs. STEM annular dark-field images of the sample (left) from the first beam exposure, where several lattice contrasts are resolved, and (right) from a subsequent scan, where no atomic contrasts can be seen anymore and carbon contamination becomes problematic. We also noticed the disappearance of the bright spheres (supposedly metallic Pb) originally decorating the platelets. The acquisition was performed at a 20 pA probe current and a 2 µs per pixel dwell time.

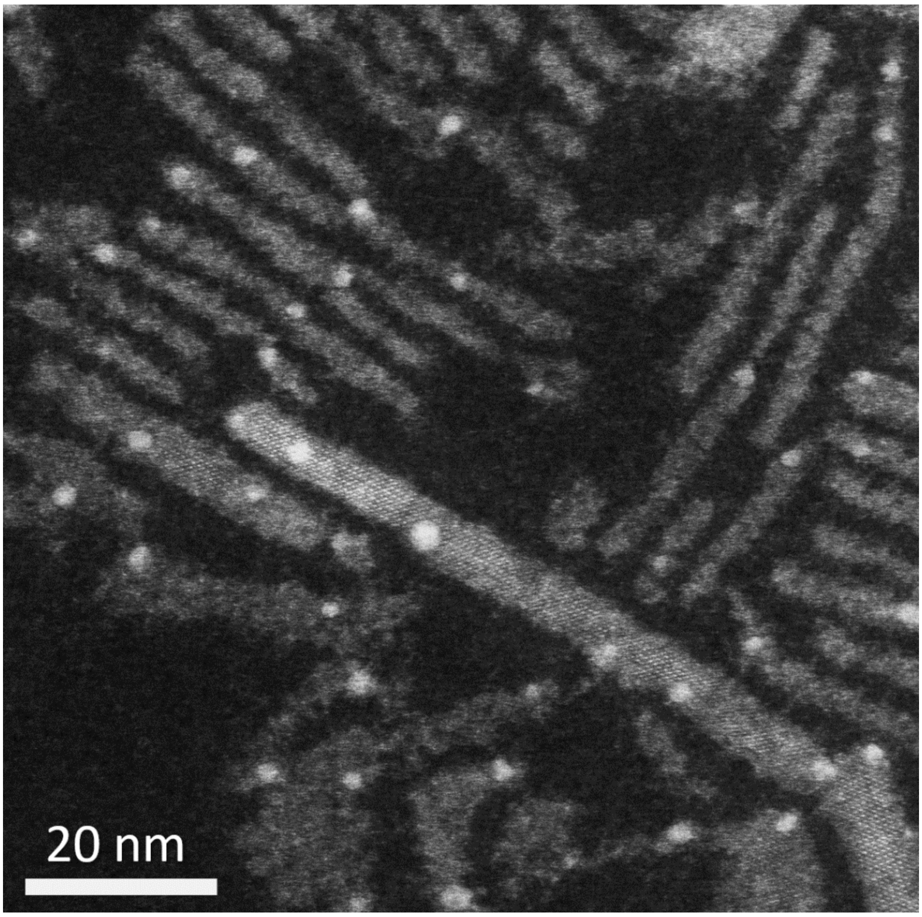

Figure S3: STEM annular dark-field image of the 3 ML NPLs sample after a few scans using a 72 pA probe current and a 2 µs dwell time. Thicker platelets, apparently 6 ML, still feature clear atomic lattice contrasts, while the thinner platelets are obviously damaged, which is understood as improved beam resistance with increasing thickness.

TEM diffraction analysis: 4D-STEM nanoprobe diffraction

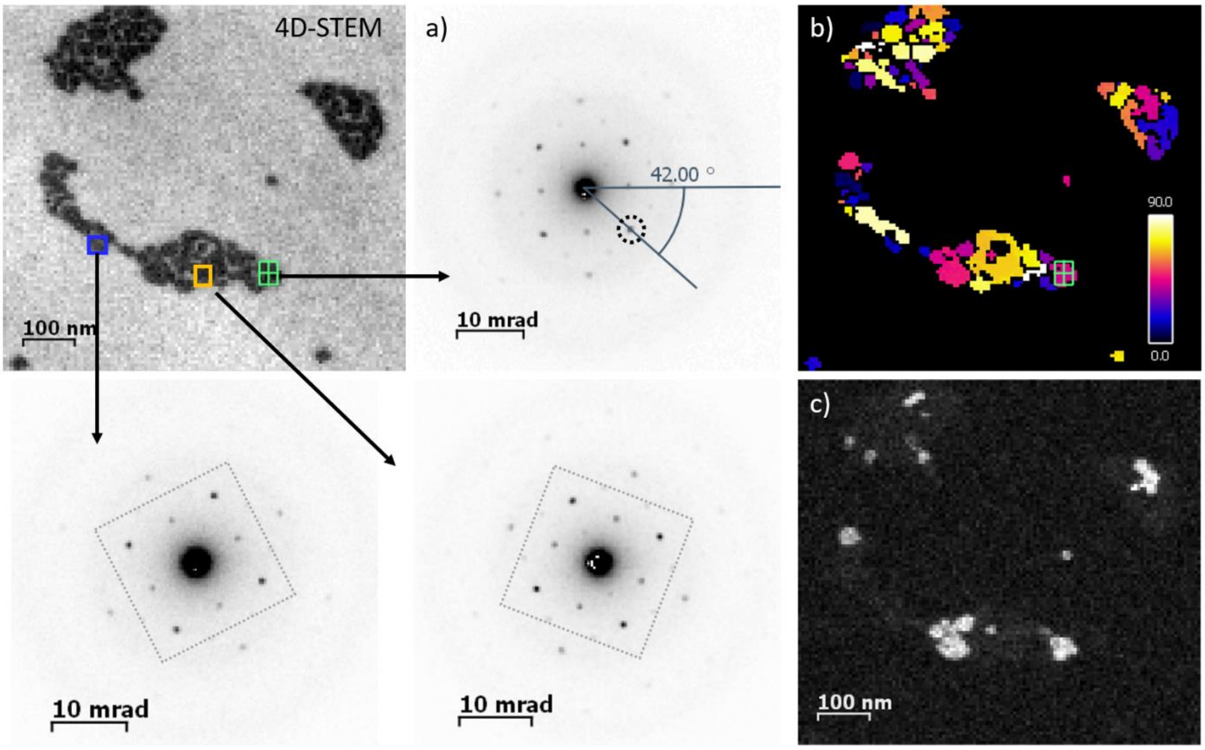

Figure S4: Nano-diffraction 4D-STEM map of the 2 ML sample, where each pixel contains a diffraction pattern. (Top-left) Reconstructed bright-field overview image containing three colored rectangles used to integrate the diffraction signal and display the respective diffraction patterns. The diffraction patterns observed across the different samples are all extremely similar but show random in-plane rotations. Due to the high number of patterns (>10,000 per map), we developed and employed simple scripts to process them and efficiently inspect the dataset. The operations consist of centering the DP and extracting the polar coordinates of the brightest spot from the lower-right detector quadrant. Two maps are drawn from this: a distance map, which represents the distance of the spot relative to the center of the DP, and an angular map, which represents the spot rotation relative to the horizontal line (as illustrated in (a)). The distance maps can highlight local crystalline variations (e.g., changes in interplanar lattice spacing) that would require further manual inspection (not shown here), while the angular maps (b) reveal the platelets' in-plane rotation, where a similar color corresponds to a similar platelet rotation. The benefits of such data processing are enhanced detection of possible NPL texture and a clearer view of the crystal domain size and shape. (c) is a reconstructed DF image from the 4D-STEM dataset using a small virtual aperture represented by the dotted circle in (a). The virtual aperture is placed around 42 degrees (e.g., pink on the color scale in (b)).

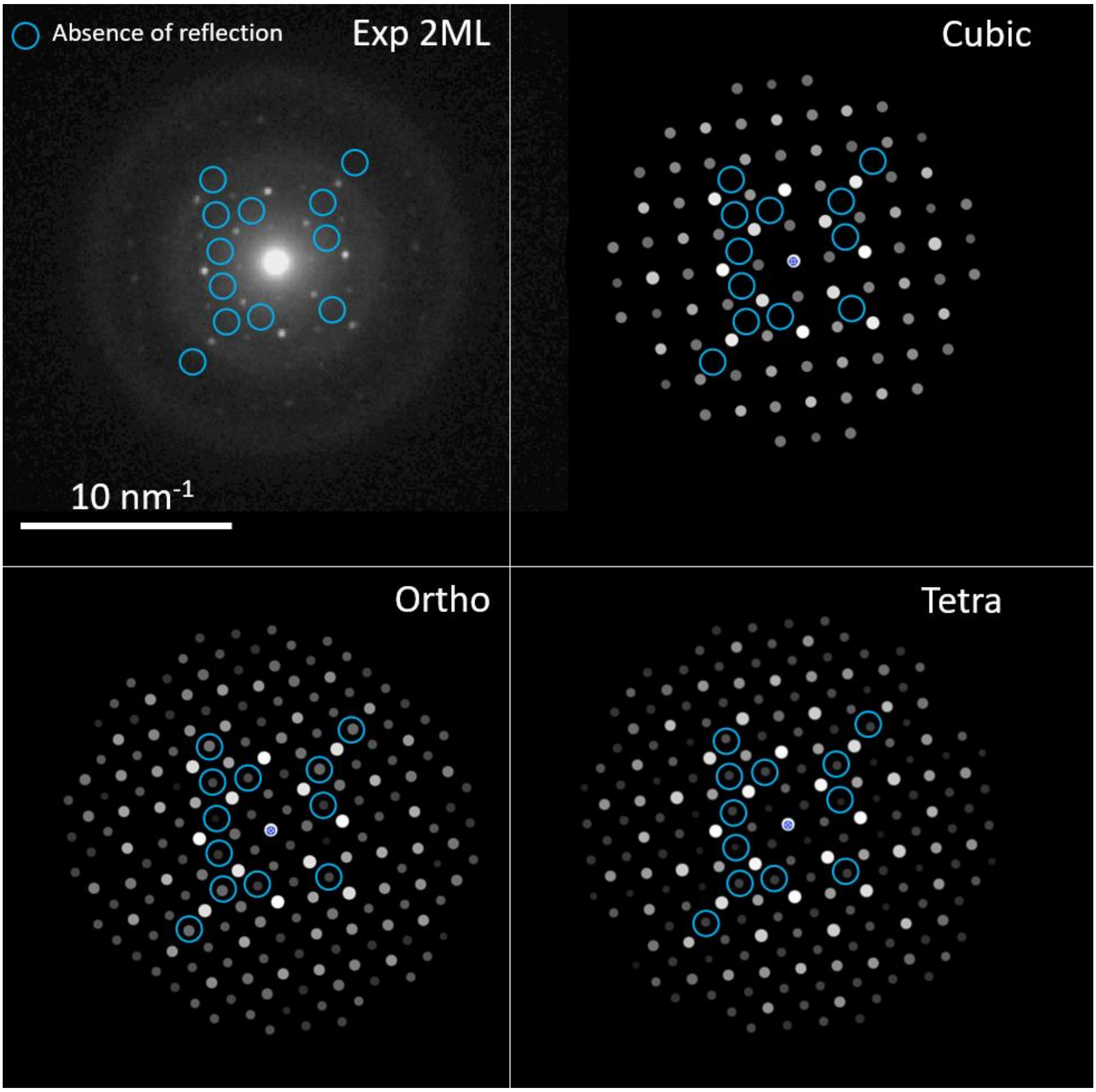

Figure S5: STEM nano-diffraction extracted from the 4D-STEM dataset of the 2 ML NPLs acquired with a 5 ms dwell time. (Top-left) Typical experimental DP. The blue circles show the absence of reflections. Experimental patterns are recalibrated using an AuPd standard acquired under the same conditions. (Top-right, bottom-left, and bottom-right) Simulated diffraction patterns using JEMS software for a [001]-oriented crystal in the cubic, orthorhombic, and tetragonal phases, respectively. These patterns are scaled using the AuPd reference and rotated to match the experimental one. The same blue circles are overlaid to aid readability.

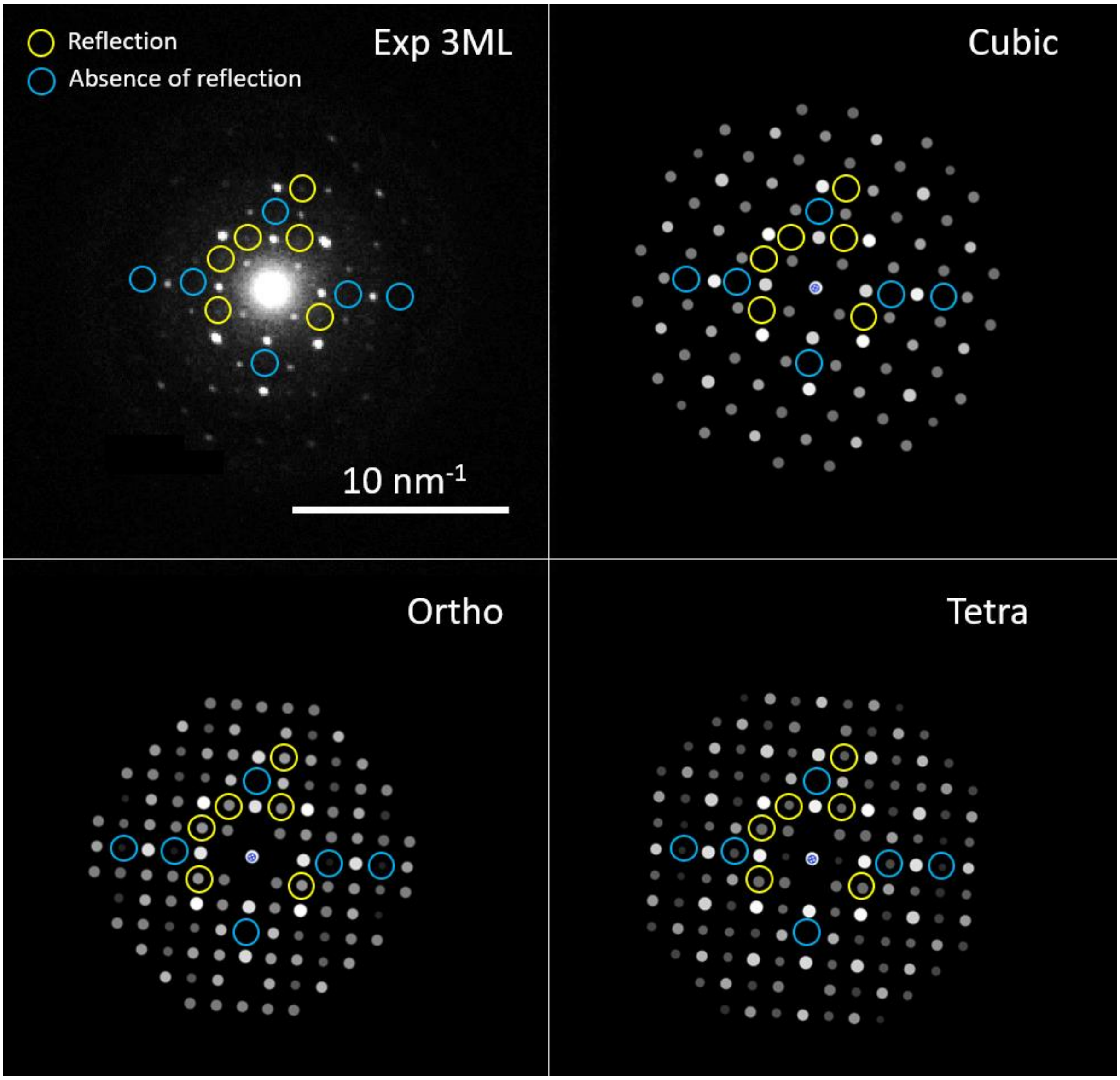

Figure S6: STEM nano-diffraction extracted from the 4D-STEM dataset of the 3 ML NPLs acquired with a 5 ms dwell time. (Top-left) Typical experimental DP. The yellow circles show the presence of reflections, and the blue circles indicate their absence. Experimental patterns are recalibrated using an AuPd standard acquired under the same conditions. (Top-right, bottom-left, and bottom-right) Simulated diffraction patterns using JEMS software for a [001]-oriented crystal in the cubic, orthorhombic, and tetragonal phases, respectively. These patterns are scaled using the AuPd reference and rotated to match the experimental one. The same set of circles is overlaid to aid readability.

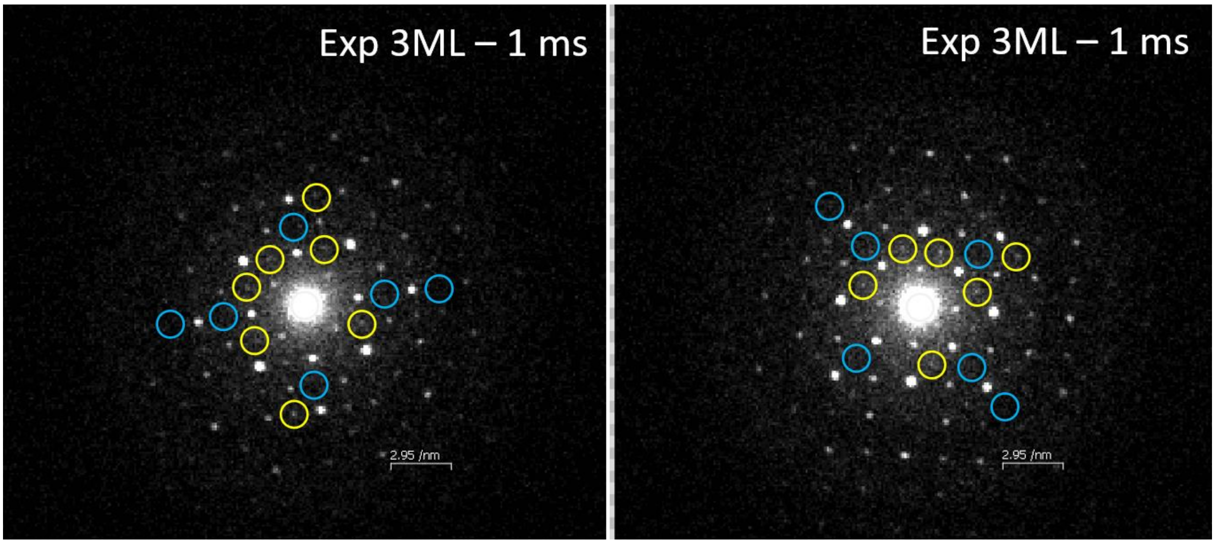

Figure S7: Two typical STEM nano-diffraction patterns extracted from the 4D-STEM dataset of the 3 ML NPLs acquired with a 1 ms dwell time. The yellow circles show the presence of reflections, and the blue circles indicate their absence. Extra reflections corresponding to the orthorhombic phase can be seen in both cases.

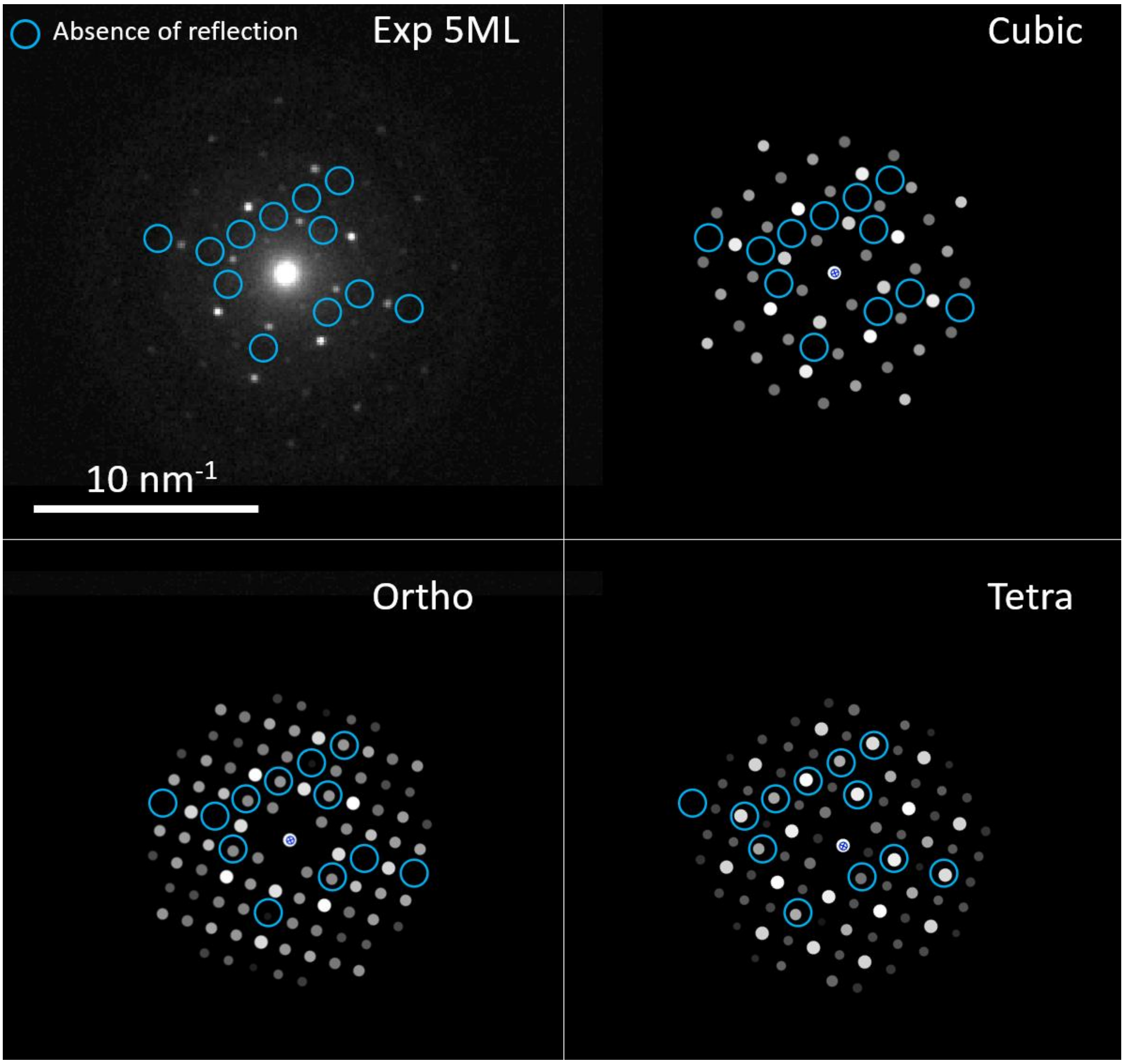

Figure S8: STEM nano-diffraction extracted from the 4D-STEM dataset of the 5 ML NPLs acquired with a 5 ms dwell time. (Top-left) Typical experimental DP. The yellow circles show the presence of reflections, and the blue circles indicate their absence. Experimental patterns are recalibrated using an AuPd standard acquired under the same conditions. (Top-right, bottom-left, and bottom-right) Simulated diffraction patterns using JEMS software for a [001]-oriented crystal in the cubic, orthorhombic, and tetragonal phases, respectively. These patterns are scaled using the AuPd reference and rotated to match the experimental one. The same set of circles is overlaid to aid readability.

Raman and PL

Chemical-treatment of CsPbBr3 nanocrystals

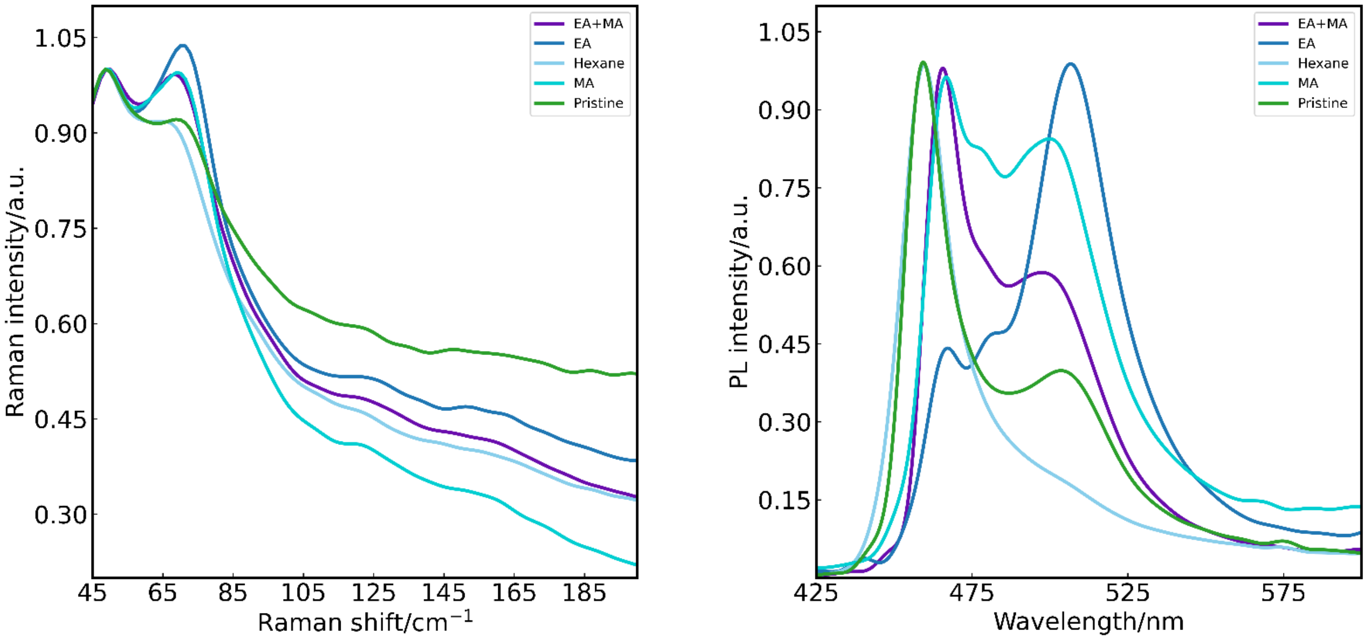

Figure S9: ((a) Raman and (b) PL spectra for pristine 3 ML NPLs and the NPLs after washing with different antisolvents. "Pristine" represents the as-prepared solution after dispersion in hexane. MA and EA stand for methyl acetate and ethyl acetate, respectively. The ratio between MA and EA in the mixed antisolvent was 0.5:0.5 mL. For the post-treatment with polar antisolvents, the precipitate was collected and re-dispersed in hexane. For hexane-based post-treatment, the supernatant was carefully collected with a syringe and used as is.

The effect of post-treatment on Raman and photoluminescence (PL) was also examined (Figure S9). Purification by hexane and centrifugation removed larger NPLs, altering both Raman and PL spectra. In contrast, polar antisolvents such as ethyl acetate and methyl acetate promoted the formation or growth of larger NPLs, as confirmed by Raman and PL, underscoring the significant influence of solvent choice on NPL stability and aggregation. In addition, the increase in the ratio between Ag and B1g prove the validation of our model upon the formation of higher number of monolayer due to aggregation.

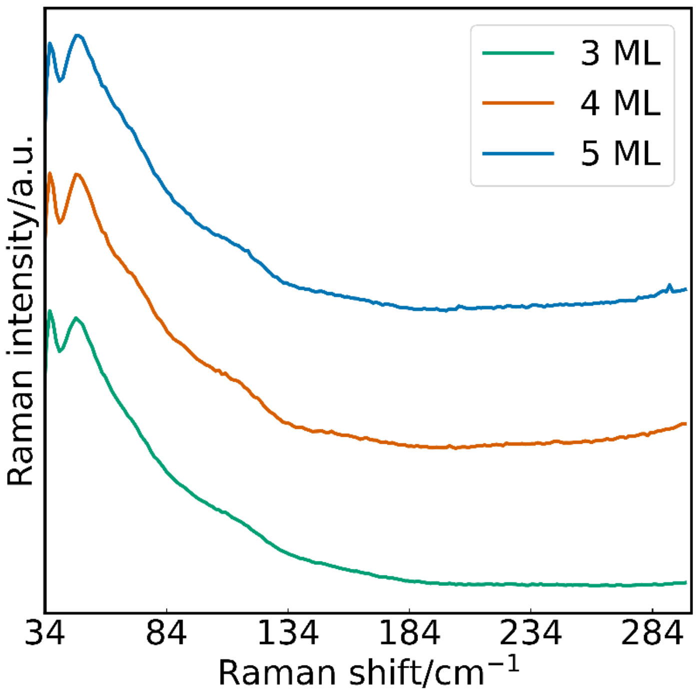

Figure S10: Raman spectrum of 3–5 ML CsPbI3. The spectra were collected under the same acquisition parameters as the bromide-based NPLs. The peak positions are shifted due to the difference in size between I and Br atoms, with the larger atomic radius causing a red shift because of changes in the polarizability domain.

We extended our analysis to iodine-based perovskite nanoplatelets (Figure S10). Like the bromide systems, we observed that the ratio of the in-plane and out-of-plane vibrational peaks changes systematically with increasing the number of monolayers from 3 to 5. These symmetry-driven trends confirm that the mechanism governing the vibrational behaviour is generic to layered halide perovskites. The $CsPbI_3$ NPls were prepared using a simple halide exchange method. The PbI_2 precursor solution was prepared separately by dissolving 0.1 mmol of $PbBr_2$ along with 100 µL each of oleylamine and oleic acid in 10 mL of toluene at 100 °C. Then, 100 µL was injected to 100 µL $CsPbBr_3$ NPls nd stirred until a complete halide exchange. The $CsPbI_3$ NPls then were deposited on a glass substrate, and the Raman spectra were acquired under the same parameter and condition of the bromide counterpart.

Symmetrical parameters calculations

To evaluate the symmetry of spectral features, we implemented a quantitative peak shape analysis. Each peak was isolated within a selected interval of the data, and the baseline was corrected prior to analysis. For baseline estimation, both linear and low-order polynomial fits of different orders were tested; the linear correction was found to best represent the real data and was therefore used in the reported Raman results. For the XRD peaks, where baseline contributions were negligible, no background correction was applied. The peak maximum intensity (y_{peak}) and its corresponding position (x_{peak}) were determined, and the half-maximum intensity was used to locate the left (x_{left}) and right (x_{right}) crossing points of the peak profile by linear interpolation. The left half-width was defined as $H_L = x_{peak} - x_{left}$, and the right half-width as $H_R = x_{right} - x_{peak}$. The full width at half maximum (FWHM) was calculated as HL+HR. The symmetry factor was defined as S = HR/HL, such that S=1 corresponds to a perfectly symmetric peak, S>1 indicates a broader right-hand side (right-skewed or tailing peak), and S<1 indicates a broader left-hand side (left-skewed peak). In addition, the statistical skewness of each peak was calculated as the normalized third central moment of the intensity distribution, using the intensity values as weights. Skewness values close to zero correspond to symmetric distributions, positive skewness indicates a longer right-hand tail, and negative skewness a longer left-hand tail.

Together, the symmetry factor provides a straightforward measure of balance at half-maximum intensity, while skewness captures the overall asymmetry of the peak profile including its tails.

This same procedure was applied consistently to both Raman and XRD data, with the only difference being the treatment of the background. For transparency and reproducibility, we provide a general MATLAB script that illustrates the full analysis workflow, including the extraction of HL, HR, FWHM, the symmetry factor, and skewness. While the Raman and XRD analyses were performed with slightly adapted scripts (to include or exclude baseline correction as required), the shared schematic code fully represents the method and can be readily adapted by others to their own data. A schematic plot (Figure S11) is also provided to visually illustrate how the quantities HL, HR, FWHM, and the symmetry factor are determined.

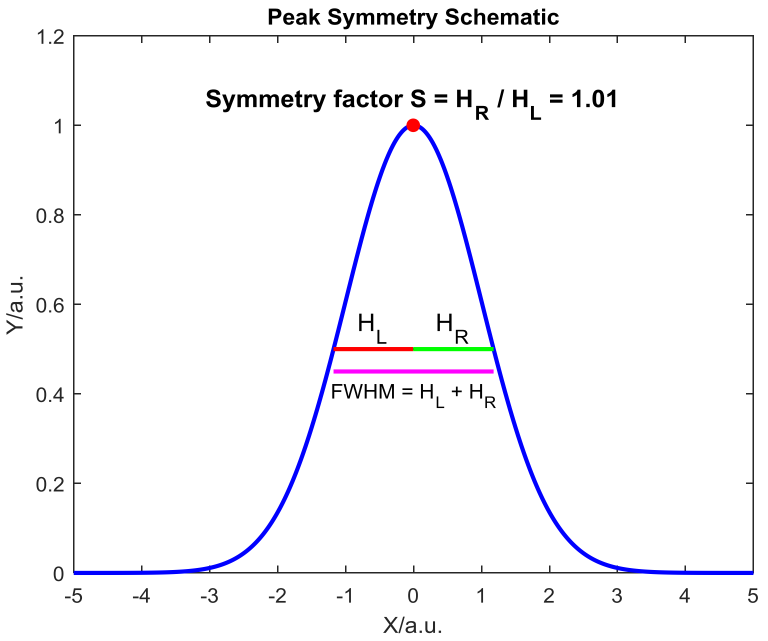

Figure S11: Schematic plot generated from a synthetic Gaussian peak, illustrating the general peak profile used in the MATLAB code provided below.

Code for the calculation of HL, HR, FWHM, skewness, and symmetry factor

```
% Code for the calculation of HL, HR, FWHM, skewness, and symmetry factor

clear; close all; clc;

% data generation
x = linspace(-5,5,1000);
y = exp(-(x.^2)/2);  % Gaussian
y = y / max(y);      % Normalize to 1

% Peak position, max, and half max
[ypeak, idxPeak] = max(y);
xpeak = x(idxPeak);
halfMax = ypeak / 2;

% Interpolation for left and right crossing
% The fix ensures unique y values and aligned x. In real data, we do not need the
fix lines
xLeft = interp1(y(1:idxPeak), x(1:idxPeak), halfMax);
```

```
[yRight, ia] = unique(y(idxPeak:end));
xRightVals = x(idxPeak:end);
xRightVals = xRightVals(ia);
xRight = interp1(yRight, xRightVals, halfMax);

% Half widths from both sides
HL = xpeak - xLeft;
HR = xRight - xpeak;
FWHM = HL + HR;
S = HR / HL;

% Plot
figure('Color','w'); hold on;
plot(x, y, 'b-', 'LineWidth', 2);
plot(xpeak, ypeak, 'ro', 'MarkerFaceColor','r');

% Plot half maximum line % Add HL and HR arrows % Mark FWHM

plot([xLeft xRight], [halfMax halfMax], 'k--', 'LineWidth', 1);
plot([xLeft xpeak], [halfMax halfMax], 'r-', 'LineWidth', 2);
plot([xpeak xRight], [halfMax halfMax], 'g-', 'LineWidth', 2);
text((xLeft+xpeak)/2, halfMax+0.05, 'H_L', 'FontSize',12,
'HorizontalAlignment','center');
text((xRight+xpeak)/2, halfMax+0.05, 'H_R', 'FontSize',12,
'HorizontalAlignment','center');

plot([xLeft xRight], [halfMax-0.05 halfMax-0.05], 'm-', 'LineWidth', 2);
text(xpeak, halfMax-0.1, 'FWHM = H_L + H_R', 'FontSize',10,
'HorizontalAlignment','center');

% Labels
xlabel('X/a.u.'); ylabel('Y/a.u.');
title('Peak Symmetry Schematic');
ylim([0 1.2]);

% Display symmetry factor in figure
text(xpeak, 1.05, sprintf('Symmetry factor S = H_R / H_L = %.2f',S), ...
    'FontSize',12, 'HorizontalAlignment','center', 'FontWeight','bold');

box on;
```

Computational method

Structural Considerations and Computational method

In bulk, $CsPbBr_3$ primarily exists in four distinct phases: a cubic phase (α-phase), a tetragonal phase (β-phase), and orthorhombic phases (γ-phase)[1]. Upon heating, these structures undergo phase transitions, transitioning from orthorhombic to tetragonal at approximately 380 K and from tetragonal to cubic at around 403 K[2]. Structurally, Pb^{2+} and Br^{-} ions form octahedral units that share bromine atoms, creating a network of connected octahedra across these phases. The alignment of lead bromide octahedra - differs between crystal structures, and their corresponding phase transitions are linked to phonon modes that induce octahedral tilting[3,4]. It is important to note that pure tetragonal and cubic phases do not exist locally. Instead, due to static and dynamic disorder, these materials exhibit cubic and tetragonal characteristics only over a finite length scale[1]. In this context, the choice of periodic slabs is well justified, given that the lateral dimensions of the NPLs are significantly larger than their vertical thickness, making periodic boundary conditions a suitable approximation.

The 2D models used in this study were generated by first optimizing the orthorhombic, cubic, and tetragonal bulk phases of $CsPbBr_3$**.** These optimized structures were then utilized as building blocks to construct slabs with two, three, four, and five layers (Figure S12, with structural parameters of the bulk phases summarized in Table S1). To minimize interactions between periodic images and replicate the effects of the organic components in NPL samples, a minimum vacuum layer of 20 Å (or larger, when specified) was introduced along the c-axis. Moreover, we tested for the case of n = 2, building the slab in other directions such as (101) for the orthorhombic case. However, this orientation is about 0.3 eV higher in energy per formula unit than (001). The terminations of the 2D layers were designed to eliminate dangling bonds**,** ensuring structural stability**.**

Additionally, for all 2D models, a global structure optimization was performed using the minima hopping (MH) algorithm. The approach follows the methodology of Goedecker *et al.*[5], where the search alternates between short molecular dynamics escape steps and subsequent local geometry relaxations. Each trial structure was generated from an initial configuration and propagated under finite-temperature molecular dynamics (initial T = 300K), followed by full ionic relaxation. Adaptive parameters were employed to control the exploration efficiency: the electronic energy tolerance was dynamically adjusted while the molecular dynamics temperature was modified according to acceptance or rejection criteria. This workflow enabled an efficient and unbiased exploration of the potential-energy landscape of $CsPbBr_3$ nanoplatelets, ensuring that the competing polymorphs were systematically sampled. No optimization of the lattice parameters was allowed. The case structures with the lowest energies were further employed in vibrational analysis.

Raman Calculations

Raman spectra for the nanoplatelets (slab models) were computed within the Placzek approximation [6], The modes intensities are given by:

$$I_i \propto \left(\frac{n(w_i,T)+1}{w_i}\right) {A_i}^2 \quad (1),$$

where

$$A_i = e_{out}^T R_i e_{in} \quad (2),$$

and

$$n(w,T) = \frac{1}{exp^{(\frac{\hbar w}{k_B T})} - 1} \quad (3).$$

Calculations were carried out at T = 300 K. Polarization unit vectors were defined as: xx (e_{in}= (1,0,0), e_{out} = (1,0,0)), xy (e_{in} = (1,0,0), e_{out} = (0,1,0)), and yy (e_{in} = (0,1,0), e_{out} = (0,1,0)). Raman spectra were obtained by convoluting the discrete mode intensities with a Gaussian broadening of 10 cm^{-1} (FWHM)**,** corresponding to a standard deviation of σ ≈ 4.25 cm^{-1}**,** to mimic experimental resolution.

Polarizability tensors R_i were obtained by applying finite differences along each vibrational mode (atoms are displaced by +0.005 Å and −0.005 Å) employing the python script written by Fornari and Stauffer[22]. This framework allows quantitative comparison between calculated Raman tensors and experimental polarization-resolved spectra.

The relation between the expansion of plane waves and the Raman intensities on the tetragonal phase was tested for energy cutoffs of 400 eV and 600 eV for the case formed with 2 ML and no difference is observed for the Raman spectra (Figure S14). On the other hand, Brillouin zone sampling, checked for reciprocal grids of 3x3x1 and 4x4x1, showed some level of variations on the obtained intensities with respect to the grid, though no changes in the mode frequencies were observed (Figure S15). Due to a compromise between computational time and accuracy, we selected a reciprocal grid of 3x3x1 and selected the cutoff to 400 eV, unless specifically shown.

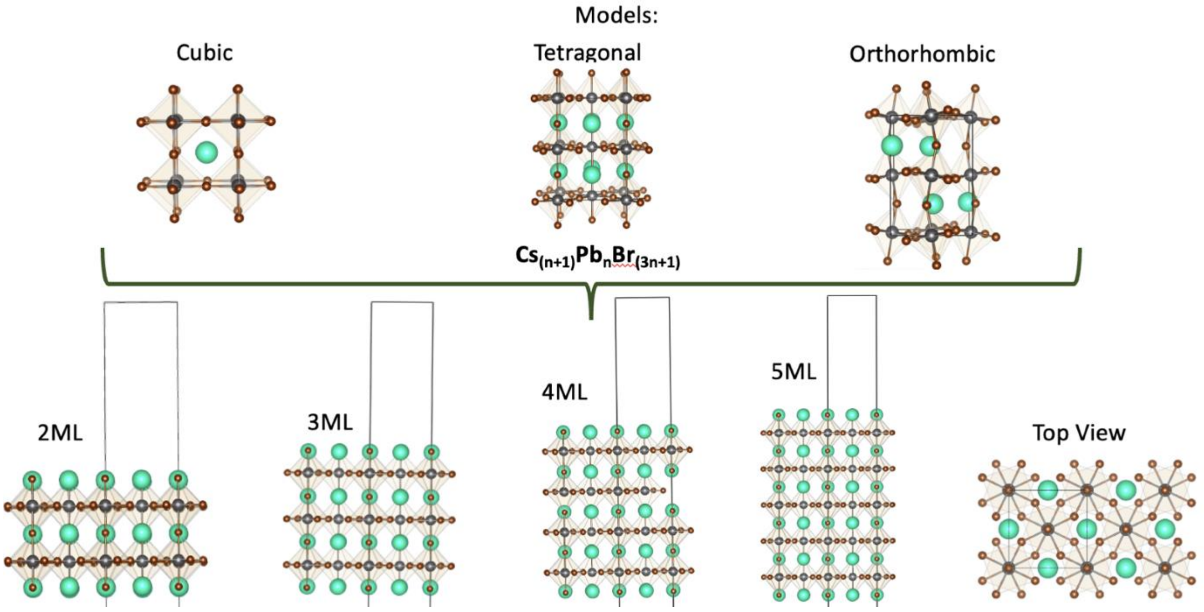

Figure S12: Atomistic models of Cs(n+1)Pb□Br_3□$_{+1}$ nanoplatelets with different layer thicknesses. Representative slab structures constructed from the bulk cubic, tetragonal, and orthorhombic phases of $CsPbBr_3$, used as starting geometries for 2D models. Each nanoplatelet (2–5 monolayers, ML) was built along the (001) direction, with surface terminations chosen to eliminate dangling bonds and a minimum vacuum spacing of 20 Å to avoid interlayer interactions. The structures illustrate the thickness-dependent dimensional confinement in the Cs(n+1)Pb□Br_3□$_{+1}$ series and the increasing number of internal $PbBr_6$ octahedral layers with n.

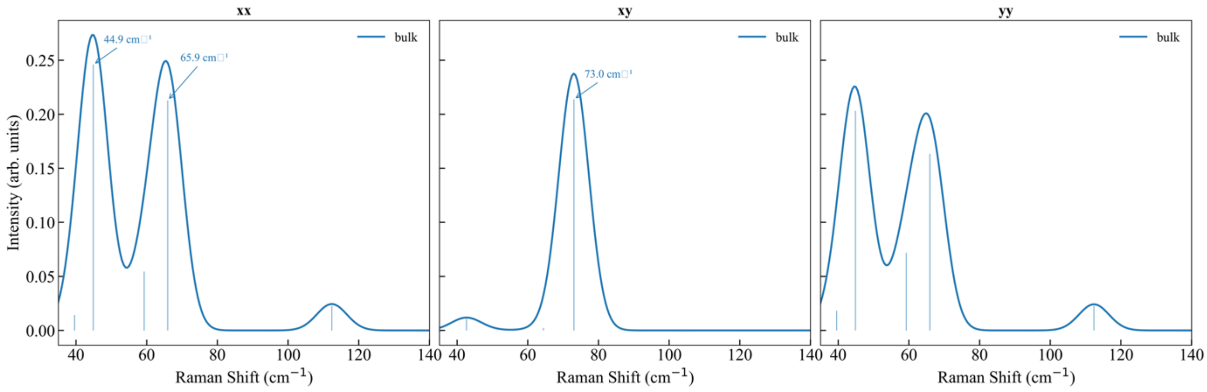

Figure S13: Polarization-resolved Raman spectra of bulk orthorhombic $CsPbBr_3$. Calculated Raman spectra for the orthorhombic $CsPbBr_3$ phase (Pnma) under three polarization configurations: xx (parallel), xy (crossed), and yy (parallel). The spectra reveal distinct selection-rule–driven activity of the A_{1g} and B_{1g} vibrational modes. In the xx and yy geometries, the A_{1g} modes dominate due to the diagonal components of the polarizability tensor, while in the xy configuration, the B_{1g} mode near 73 cm^{-1} is enhanced, reflecting activation of off-diagonal tensor elements. Vertical markers indicate the corresponding mode positions from the DFT calculations. Moreover, a Gaussian broadening of 10 cm^{-1} (FWHM) was employed together with a temperature of 300 K.

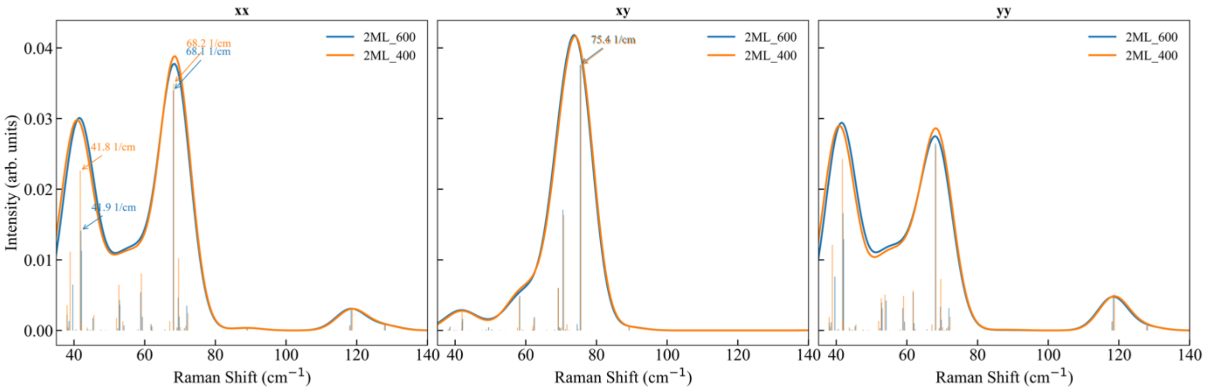

Figure S14: Calculated Raman spectra of the 2 ML $CsPbBr_3$ nanoplatelet with different plane-wave cutoff energies. Comparison of Raman spectra for 2 ML $CsPbBr_3$ slabs in the orthorhombic phase computed at the PBE–SOC level of theory. The close similarity between spectra obtained with different plane-wave cutoff energies confirms that the main vibrational features are well converged and remain unaffected by the chosen energy threshold. Vertical markers indicate the corresponding mode positions from the DFT calculations. Moreover, a Gaussian broadening of 10 cm^{-1} (FWHM) was employed together with a temperature of 300 K.

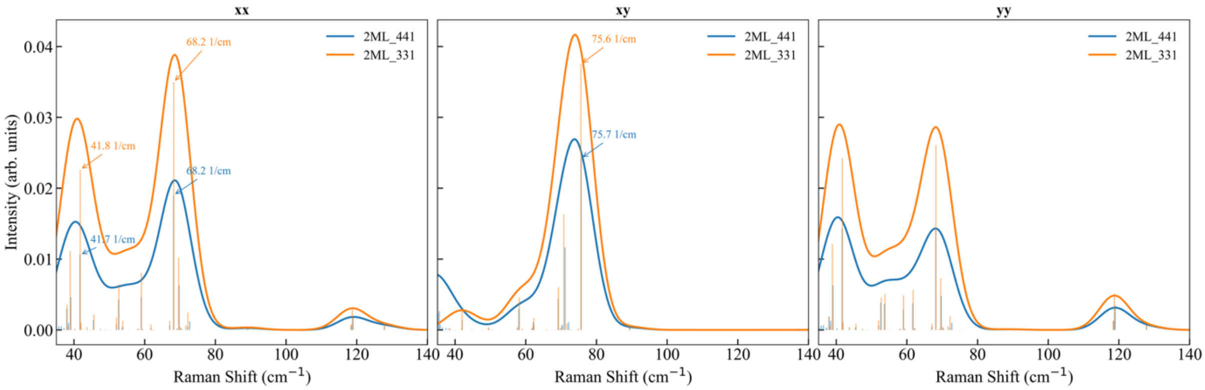

Figure S15: Calculated Raman spectra of the 2 ML $CsPbBr_3$ nanoplatelet using different Brillouin-zone sampling grids. Comparison of Raman spectra for 2 ML $CsPbBr_3$ slabs in the orthorhombic phase computed at the PBE–SOC level of theory using 4×4×1 and 3×3×1 k-point meshes. The overall spectral profiles and mode positions remain consistent across grids, confirming convergence of the phonon frequencies and Raman intensities with respect to Brillouin-zone sampling. Although slight differences in relative peak intensities are observed, the overall shape of the spectra remains unchanged for both grids, indicating robust spectral behavior against k-point sampling variations. Vertical markers indicate the corresponding mode positions from the DFT calculations. Moreover, a Gaussian broadening of 10 cm^{-1} (FWHM) was employed together with a temperature of 300 K.

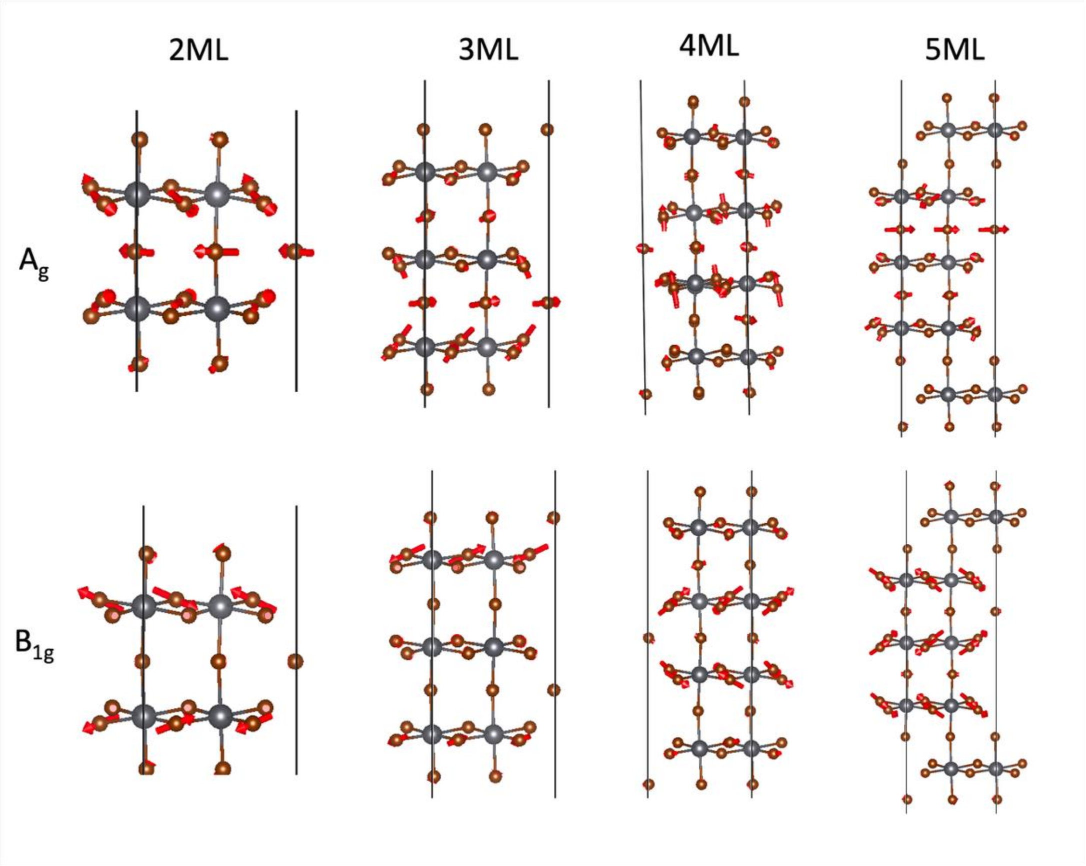

Figure S16: Thickness-dependent vibrational eigenmodes of $CsPbBr_3$ nanoplatelets. DFT-calculated atomic displacement patterns for the Ag (top row – around 45 cm^{-1}) and B_1g (bottom row) Raman-active modes in $Cs(n+1)Pb_{\square}Br_{3\square+1}$ nanoplatelets with 2–5 monolayers (MLs). Red arrows indicate the direction and relative magnitude of atomic vibrations. The Ag mode involves Pb–Br–Pb bond bending that couples with out-of-plane displacements, whereas the B_1g mode corresponds to Pb–Br–Pb vibrations primarily confined to the in-plane direction. As the Ag mode couples with the out-of-plane direction, symmetry breaking leads to a stronger effect on the Raman intensities for this mode compared to the B_1g mode, hence explaining the experimentally observed enhancement of B_1g Raman intensity with thickness relative to the intensity change of the Ag mode.

Table 1: Optimized lattice parameters of bulk $CsPbBr_3$ phases used for constructing nanoplatelet models. Calculated lattice constants (a, b, c) and angles (α, β, γ) for the orthorhombic, cubic-distorted, and tetragonal phases of $CsPbBr_3$ obtained from DFT geometry optimization. The results show the expected structural evolution from the low-symmetry orthorhombic phase to the higher-symmetry tetragonal and cubic forms, which serve as the structural templates for the corresponding nanoplatelet models.

	a (Å)	b (Å)	c (Å)	alpha	beta	gamma
Orthorhombic	8.2	8.5	11.9	90.0	90.0	90.0
Cubic Distorted	11.9	11.9	11.9	91.7	90.0	90.0
Tetragonal	11.7	11.7	12.1	90.0	90.0	90.0

Bibliography

(1) Hoffman, A. E. J.; Saha, R. A.; Borgmans, S.; Puech, P.; Braeckevelt, T.; Roeffaers, M. B. J.; Steele, J. A.; Hofkens, J.; Van Speybroeck, V. Understanding the Phase Transition Mechanism in the Lead Halide Perovskite CsPbBr3 via Theoretical and Experimental GIWAXS and Raman Spectroscopy. *APL Mater.* **2023**, *11* (4), 041124. https://doi.org/10.1063/5.0144344.

(2) Mannino, G.; Deretzis, I.; Smecca, E.; La Magna, A.; Alberti, A.; Ceratti, D.; Cahen, D. Temperature-Dependent Optical Band Gap in CsPbBr3, MAPbBr3, and FAPbBr3 Single Crystals. *J. Phys. Chem. Lett.* **2020**, *11* (7), 2490–2496. https://doi.org/10.1021/acs.jpclett.0c00295.

(3) Bertolotti, F.; Protesescu, L.; Kovalenko, M. V.; Yakunin, S.; Cervellino, A.; Billinge, S. J. L.; Terban, M. W.; Pedersen, J. S.; Masciocchi, N.; Guagliardi, A. Coherent Nanotwins and Dynamic Disorder in Cesium Lead Halide Perovskite Nanocrystals. *ACS Nano* **2017**, *11* (4), 3819–3831. https://doi.org/10.1021/acsnano.7b00017.

(4) Bechtel, J. S.; Thomas, J. C.; Van der Ven, A. Finite-Temperature Simulation of Anharmonicity and Octahedral Tilting Transitions in Halide Perovskites. *Phys. Rev. Mater.* **2019**, *3* (11), 113605. https://doi.org/10.1103/PhysRevMaterials.3.113605.

(5) Goedecker, S. Minima Hopping: An Efficient Search Method for the Global Minimum of the Potential Energy Surface of Complex Molecular Systems. *J. Chem. Phys.* **2004**, *120* (21), 9911–9917. https://doi.org/10.1063/1.1724816.

(6) Porezag, D.; Pederson, M. R. Infrared Intensities and Raman-Scattering Activities within Density-Functional Theory. *Phys. Rev. B* **1996**, *54* (11), 7830–7836. https://doi.org/10.1103/PhysRevB.54.7830.
